# Synthesis and Excimer Formation Properties of Electroactive Polyamides Incorporated with 4,5-Diphenoxypyrene Units

**DOI:** 10.3390/polym14020261

**Published:** 2022-01-09

**Authors:** Shih-Hsuan Chen, Huai-Sheng Chin, Yu-Ruei Kung

**Affiliations:** Department of Chemical Engineering and Biotechnology, Tatung University, Taipei City 104327, Taiwan; sean95123w@gmail.com (S.-H.C.); kim021324@gmail.com (H.-S.C.)

**Keywords:** high performance polymers, polyamides, electroactive polymers, pyrene, excimer emission

## Abstract

A new dietherpyrene-cored diamine monomer, namely, 4,5-bis(4-aminophenoxy)pyrene, was successful synthesized and formed a series of electroactive polyamides with an aryloxy linkage in a polymer main chain and bearing pyrene chromophore as a pendent group using conventional one-pot polycondensation reactions with commercial aromatic/aliphatic dicarboxylic acids. The resulting polyamides exhibited good solubility in polar organic solvents and, further, can be made into transparent films. They had appropriate levels of thermal stability with moderately high glass-transition values. The dilute NMP solutions of these polyamides exhibited pyrene characteristic fluorescence and also showed a remarkable additional excimer emission peak centered at 475 nm. Electrochemical studies of these polymer films showed that these polyamides have both p- and n-dopable states as a result of the formation of radical cations and anions of the electroactive pyrene moieties.

## 1. Introduction

Since the 1930s, the development of polyamide technology established many of the principles and practices for polymerization in general, laying the deeply impact groundwork for the great array of materials that have followed. Traditional aromatic polyamides are well-known as high-performance polymer materials; they have outstanding thermal, mechanical and electrical properties as well as excellent chemical resistance [1,2,3]. However, most of them are insoluble in most organic solvents due to the rigidity of the molecular backbone, together with strong intermolecular interactions and lead to a high melting point or softening temperature. These characteristics usually make them difficult to process; therefore, their applications are limited in certain areas [4,5]. In order to overcome these limitations, many studies have been carried out to improve the processing characteristics of these polymers, while retaining the original excellent properties [6,7,8,9,10]. For overcoming the related drawbacks, it is basically necessary to design and develop a new monomer via structure modification. For example, the facile strategies of adding a flexible ether linkage connection (-O-) to the polymer backbone, or introducing a large aromatic ring, non-coplanar or asymmetric unit has been proven to increase organic solubility without sacrificing its original excellent performance method [11,12,13,14,15,16].

Pyrene is a polycyclic aromatic hydrocarbon molecule formed by four fused benzene rings. Its attractive features include its functionalization, delaying the appearance of fluorescence, obvious solvent discoloration and a high tendency to form active excimer formations [17,18,19,20]. Pyrene and its derived compounds have been proverbially studied and applied as a fluorescent detection sensor [21,22,23,24]. In recent years, due to the outstanding light-emitting properties associated with the high charge carrier mobility of pyrene derivatives, the application for organic electronic materials in the context of organic light-emitting diode devices (OLED), such as pyrene derivatives [25,26], polymers [27], starburst [28] and dendrimers [29,30], have been widely reported. Resulting in red shift emission and accompanied by a significant reduction in fluorescence efficiency, 1, 3, 6, 8-tetraphenylpyrene (TPPy) has a fluorescence efficiency of up to 90% in solution [31], but the components made of TPPy solid film only have an external fluorescence efficiency of 0.5% in the electroluminescence devices [32]; therefore, the use of pyrene as a luminescent material in OLED applications is significantly restricted. Fortunately, through molecular structure design, the tight packing/fluorescence quenching phenomenon in pyrene-based materials can be reduced or controlled. For example, 1, 3, 6, 8- or 1, 3, 5, 9-tetra substituted pyrene can successfully prevent π-stacking in small molecules, which can produce blue light with high efficiency both in solution and solid states [33,34]. Therefore, many studies have introduced aromatic groups with π-stacking steric barriers into the molecular structure of pyrene, which can effectively improve the luminous efficiency of such compounds in the solid state [35,36]. However, there is not much research and literature on 4, 5, 9, 10-tetra-substituted pyrene [37,38,39,40,41,42]. The main obstacle to the synthesis is that the electrophilic substitution of pyrene preferentially takes place at the 1, 3, 6, 8 positions (non-K-region), as accessing the 4, 5, 9, 10 positions (K-region) is much more difficult. Indeed, functionalization at the K region of pyrene often requires multi-step syntheses with low overall yields. In the past 20 years, there has been no research focus on attaching phenoxy groups to pyrene’s K region and further via functionalization into reactive groups.

For the continuous development of traditional high-performance polymers such as polyamides and endowing them with new optoelectronic functionality, we tried to introduce opto-and redox active pyrene into its polymer backbone. Due to the attractive fluorescent and redox active properties of pyrene, we incorporated the phenoxy linkage to the K region of pyrene, further subjoined reactive diamine as a functional group, and finally, used polycondensation via one-stepwise with commercially available dicarboxylic acids to produce pyrenyl-bearing polyamides with optoelectronic activity. The built-in properties, such as solubility, film-forming capability and thermally behaviors, of the prepared dietherpyrene-based polyamide will be investigated. Their optical and electrochemical properties will also be widely studied, and according to optoelectronic properties to find the suitable applications in the light-emitting, hole-transporting and redox flow batteries fields.

## 2. Materials and Methods

### 2.1. Materials

Pyrene (Acros Organics, Geel, Belgium), ruthenium(III) chloride hydrate (RuCl_3_∙xH_2_O) (Alfa Aesar, Stoughton, MA, USA), sodium metaperiodate (NaIO_4_) (Alfa Aesar, Stoughton, MA, USA), sodium sulfate (Na_2_SO_4_) (Showa Chemical, Minato-ku, Tokyo, Japan), anhydrous magnesium sulfate (MgSO_4_) (Showa Chemical, Minato-ku, Tokyo, Japan), sodium dithionite (Na_2_S_2_O_4_) (Aencore, Box Hill, Australia), tetra-*N*-butylammonium bromide (Alfa Aesar, Stoughton, MA, USA), sodium hydroxide (Thermo Fisher Scientific, Waltham, MA, USA), 4-fluoronitrobenzene (Alfa Aesar, Stoughton, MA, USA), 10% palladium on carbon (Pd/C) (Alfa Aesar, Stoughton, MA, USA), calcium chloride (CaCl_2_) (Showa Chemical, Minato-ku, Tokyo, Japan) and triphenyl phosphite (TPP) (Alfa Aesar, Stoughton, MA, USA) were used without further purification. Dichloromethane (CH_2_C1_2_) (ECHO chemical, Miaoli, Taiwan), acetonitrile (CH_3_CN) (Aencore, Box Hill, Australia), tetrahydrofuran (THF) (ECHO chemical, Miaoli, Taiwan), acetic acid glacial (ECHO chemical, Miaoli, Taiwan), *N*,*N*-dimethylacetamide (DMAc) (TEDIA, Fairfield, OH, USA), pyridine (Py) (Thermo Fisher Scientific, Waltham, MA, USA) and *N*-methyl-2-pyrrolidone (NMP) (TEDIA, Fairfield, OH, USA) were used as received from the local supplier. All other ingredients were used as received from suppliers/producers. The commercially available aromatic dicarboxylic acids, such as 1,4-cyclohexanedicarboxylic acid (**4a**) (TCI chemical, Saitama, Japan), terephthalic acid (**4b**) (TCI chemical, Saitama, Japan), 4,4′-dicarboxydiphenyl ether (**4c**) (TCI chemical, Saitama, Japan), 4,4’-dicarboxydiphenyl sulfone (**4d**) (TCI chemical, Saitama, Japan) and 2,2-bis(4-carboxyphenyl)hexafluoropropane (**4e**) (TCI chemical, Saitama, Japan), were used as received from the local supplier, ECHO chemical, Taiwan. The synthetic details and analysis of pyrene-4,5-dione (**1**), 4,5-bis(4-nitrophenoxy)pyrene (**2**), 4,5-bis(4-aminophenoxy)pyrene (**3**), 4,5-di(4-benzamidophenoxy)pyrene (**M1**), 4,5-di(4-cyclohexanecarboxamidophenoxy)pyrene (**M2**) and polyamides were summarized in the Supplementary Materials.

### 2.2. Methods

Infrared (IR) spectra were conducted on the spectrum GX FTIR system (PerkinElmer, Waltham, MA, USA). The proton and carbon NMR spectra were gauged on a Avance III HD-600 MHz NMR (Bruker, Fremont, CA, USA) with tetramethylsilane (TMS) as standard. The inherent viscosities were measured with a Cannon–Fenske viscometer at 30 °C for an average of five times. Molecular weights (*M*_w_/*M*_n_) were obtained from gel permeation chromatography (GPC) via PU-2080 Plus HPLC Pump and RI-2031 detector system (JASCO, Hachioji, Tokyo, Japan) on the basis of a polystyrene calibrated baseline using dried THF as the fresh eluent. Wide-angle X-ray diffraction (WAXD) data were performed at ca. 25 °C on a XRD-6000 X-ray diffractometer (Shimadzu, Nakagyo-ku, Kyoto, Japan), using graphite-monochromatized and nickel-filtered Cu-Kα radiation (λ = 1.5418 Å) with the operating factor at 40 kV and 30 mA, and the scanning rate was carried out with 2 degree/min in a range of 2θ = 10~40 °. Single crystal crystallography data of a synthesized target monomer were conducted with D8 Venture Dual X-ray Single Crystal Diffractometer (Bruker, Fremont, CA, USA). Ultraviolet–visible (UV–Vis) spectra both in solutions and films were recorded on a V-530 UV/VIS Spectrophotometer (JASCO, Hachioji, Tokyo, Japan) and an 8453 UV–Visible spectrophotometer (Agilent, Santa Clara, CA, USA), respectively. Thermogravimetric analysis (TGA) was performed with a Pyris 1 TGA (PerkinElmer, Waltham, MA, USA). Measurements were carried out on approximately 3–5 mg of samples heated in flowing nitrogen or air (flow rate = 20 cm^3^/min) at a heating rate of 20 °C/min. DSC analyses were performed on a Pyris 1 DSC (PerkinElmer, Waltham, MA, USA) at a scan rate of 20 °C/min in flowing nitrogen. Electrochemistry was performed with a 6116E electrochemical analyzer (CH Instruments, Austin, TX, USA). Cyclic voltammetry was measured with the use of a three-electrode system in which ITO-coated glass (sample area about 2.0 cm^2^) was used as a working electrode and together with platinum wire as an auxiliary electrode. All potentials of samples were taken with the use of a self-made Ag/AgCl, with KCl (sat.) as a reference electrode. Ferrocene (Fc) was used as an external standard for calibration (+0.48 V vs. Ag/AgCl).

## 3. Results and Discussion

### 3.1. Synthesis of Intermediates and Monomer

**Scheme 1:**
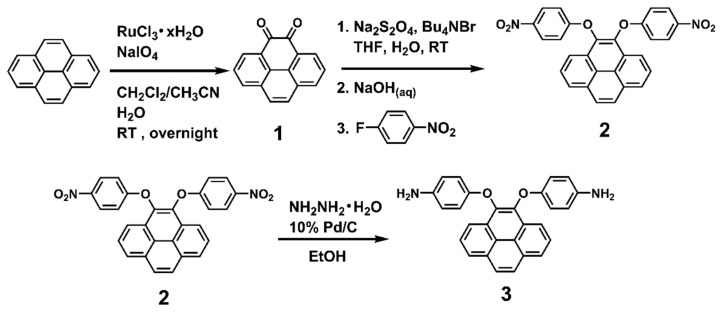
Synthetic routes to pyrene-cored diamine (**3**).

**Scheme 2:**
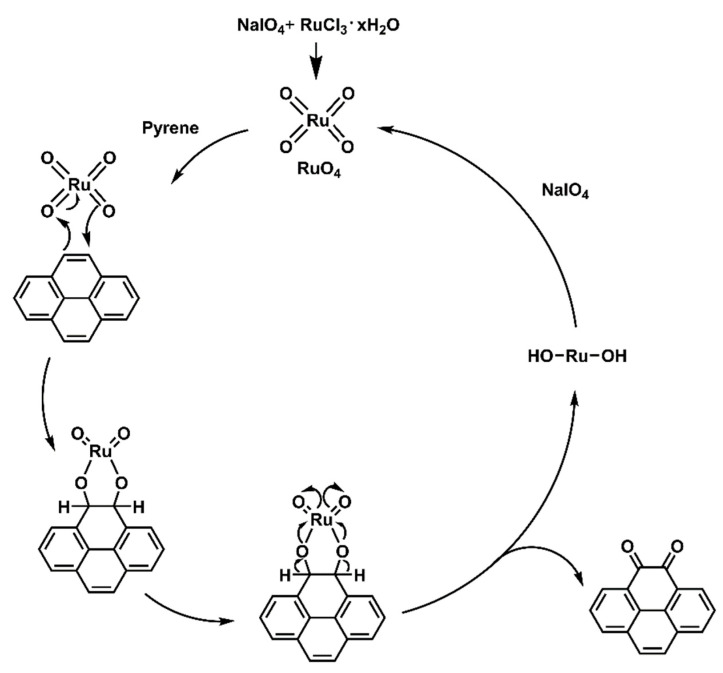
The postulated oxidation mechanism pathways of pyrene using RuCl_3_/NaIO_4_ catalytic system.

The dietherpyrene-based diamine **3** was successfully synthesized by the synthetic route outlined in Scheme 1. Pyrene-4,5-dione (**1**) was prepared by using a modified procedure from the selective K-region oxidation of pyrene that presented the stoichiometric quantities of ruthenium tetraoxide (RuO_4_) from the RuCl_3_/NaIO_4_ catalytic system in previous studies [43,44,45]. The postulated oxidation mechanism pathways from starting pyrene to pyrene-4,5-dione intermediates are shown in Scheme 2.

**Figure 1:**
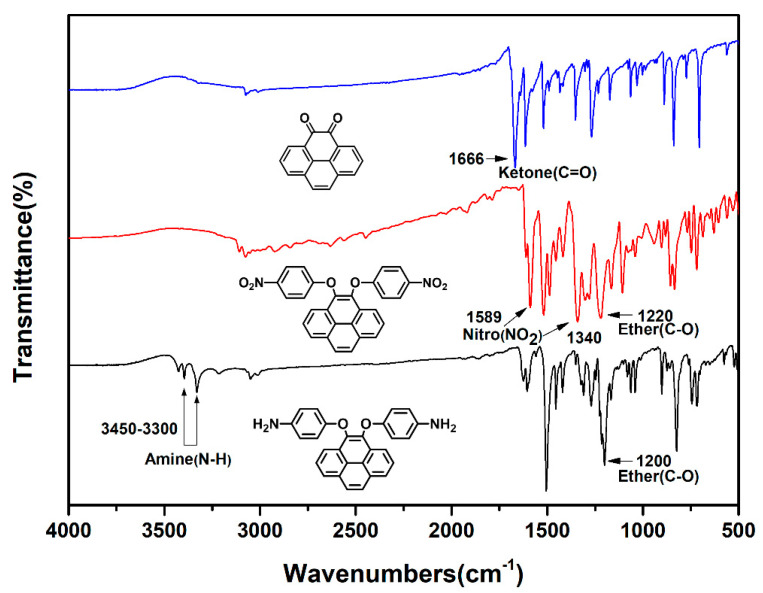
FT-IR spectra of pyrene-derived compounds and monomers **1**–**3**.

Due to high tendency oxidation from 4,5-dihydroxypyrene to pyrene-4,5-dione in atmosphere, dinitro compound (**2**) was synthesized by a one-pot direct reduction of a carbonyl group of pyrene-4,5-dione with sodium dithionite to be converted to a diol intermediate and then this was followed by the diarylation of 4,5-dihydroxypyrene with 4-fluoronitrobenzene in the presence of sodium hydroxide as the base and tetra-*n*-butylammonium bromide salt as the phase transfer catalyst [46]. The final target diamine monomer 4,5-bis(4-aminophenoxy)pyrene (**3**) was prepared by a hydrazine Pd/C-catalyzed reduction of 4,5-bis(4-nitrophenoxy)pyrene (2) in a high yield. The structures of pyrene-4,5-dione (**1**), the dinitro intermediate (**2**) and the target diamine monomer (**3**) were confirmed with IR, NMR and single crystal X-ray diffraction analysis. The FT-IR spectra of compounds **1** to **3** can be seen in Figure 1. Pyrene-4,5-dione (1) shows the characteristic absorption at 1666 cm^−1^ (ketone C = O stretch). After the reduction of the carbonyl group of (**1**) and then the following diarylation to form a bis(nitrophenoxy)pyrene unit, the ketone characteristic band disappeared and the nitro groups show the pair characteristic absorption bands at 1340 and 1589 cm^−1^ (-NO_2_ symmetric and asymmetric stretching), and a strong aryl ether band located at 1220 cm^−1^ (C-O stretching). After reduction to diamine **3**, the nitro group absorptions were eliminated, and the primary amino unit showed the typical absorption pair located at 3450–3300 cm^−1^ as a result of N-H stretching.

**Figure 2:**
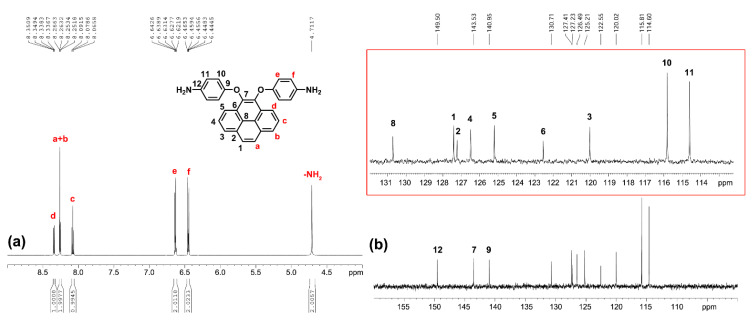
(**a**) Proton and (**b**) carbon NMR spectra (enlarge region between 110 to 132 ppm as shown in red frame) of monomer **3** in DMSO-d_6_.

**Figure 3:**
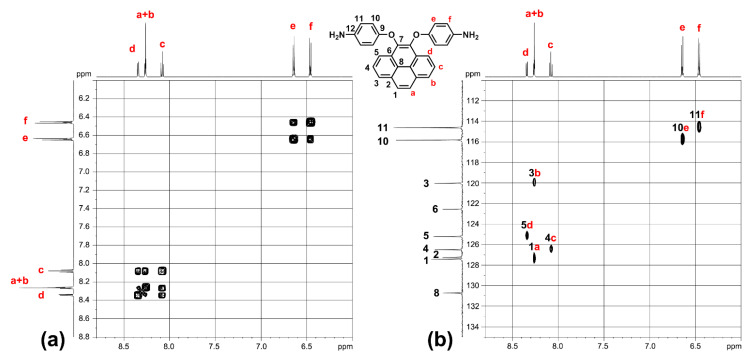
(**a**) H-H COSY and (**b**) C-H HSQC NMR spectra of target monomer 3 in DMSO-d_6_.

The ^1^H NMR, ^13^C NMR and 2D NMR spectra of the intermediate compounds **1**–**2** and the target diamine monomer **3** are illustrated systematically with fully peak assignments in Figures S1–S4 and Figure 2, respectively. The accomplished conversion of nitro units to the primary amino units was confirmed by the high-field shift of the phenylene protons and by the resonance signals at around 4.71 ppm corresponding to the amino protons. Although the symmetric substitution at the 4,5 positions of the pyrene unit, the spectra of diamine **3** are a bit complicated in one dimensional NMR. In order to fully assign all the peaks, we took two-dimensional (2D) COSY and HSQC NMR spectra, as demonstrated in Figure 3.

**Figure 4:**
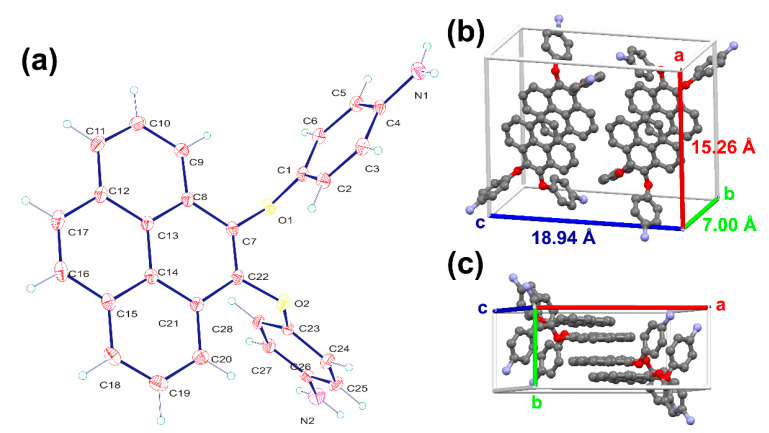
(**a**) Conclusive structure of diamine 3 using single-crystal X-ray analysis and the (**b**) front view and (**c**) side view of single-crystal lattice with cross-section profiles.

Thus, the results of all the spectroscopic and single crystal X-ray diffraction analysis suggest the expected structure of diamine **3** was successfully synthesized. The authentic structure of diamine 3 was further comprehended using single-crystal X-ray diffraction analysis. The molecular structure of pyrene-cored diamine 3 (Figure 4) shows that the pyrene and phenyl rings were not in the same plane due to a flexible ether unit as linkage. As shown in Figure 4b,c, the crystal lattice also showed the high tendency to form dimer-like planar with ca. 3.50 Å between two pyrene structures. The bulky pyrene and non-planar ether conformation was prospected to reduce the packing efficiency of polymer chains and to improve the solubility of its derived polymers. The model compounds **M1** and **M2** were also prepared from the one-pot synthesis of diamine 3 with two equivalent amounts of benzoic acid and cyclohexanecarboxylic acid, as illustrated in Scheme S1. Their IR and NMR spectroscopic data of absorption, proton and carbon signals were fully assigned, as shown in Figures S5–S10.

### 3.2. Polyamides Synthesis

**Scheme 3:**
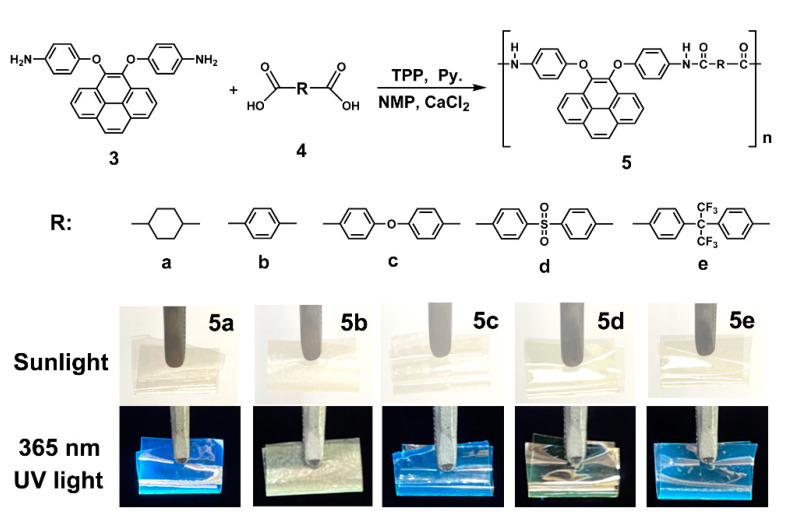
Synthesis of dietherpyrene-based polyamides and their foldable films before and after irradiated by laboratory UV lamp at wavelength of 365 nm.

On the basis of the well-established method depicted by the Yamazaki phosphorylation reaction, it is common knowledge that the triphenyl phosphite (TPP) and pyridine as an inorganic complex medium played an important role in obtaining homogenous high molecular weight polymers in the polymerization of diamines and dicarboxylic acids [47,48]. A series of novel dietherpyrene-cored structural polyamides **5a**–**5e** were synthesized from diamine 3 with various aliphatic and aromatic dicarboxylic acids (**4a**–**4e**) using the direct polycondensation reaction with Yamazaki condensing agents (Scheme 3). After the reaction, all the polymerizations were carried out in a homogeneous phase and become a transparent high-viscosity solution. When the obtaining polymer solution was poured into stirred methanol, these polymers precipitated in a strong and fiber-like constitute. The typical as-prepared sample and the foldable film of polyamide **5a** displayed a strong blue emission upon laboratory UV lamp irradiation also as shown in Scheme 3. The structures of the polyamides could be confirmed using IR and NMR spectroscopy. The collected IR spectrum for a typical polyamide **5e** in Figure S11, which indicates the featured absorption bands of the amide group at around 3295 cm^−1^ (amide N-H stretching) and 1627 cm^−1^ (amide C = O stretching). The ^1^H NMR spectra, as illustrated in Figure S11, for polyamide **5e** shows that the resonance peak emerging at 10.3 ppm clearly represents the formation of an amide linkage.

### 3.3. Basic Characterization

All the dietherpyrene-based polyamides could be casted into transparent, rollable and tough films. The WAXD studies of these film samples revealed that all the polyamides were essentially a non-crystalline domain, as shown in Figure S12. These quantitative obtained polyamides had inherent viscosities with η_inh_ values in the range of 0.41–0.70 dL/g (Table 1). All the fiber-like polymer samples could afford transparent and foldable films, as shown in Scheme 3, via common casting, demonstrating high molecular weights. THF-soluble polyamide **5e** showed a weight-average molecular weight (M_w_) of 66,000 and a number-average molecular weight (M_n_) of 53,500 with a polydispersity index (PDI) of 1.23 by GPC measurements. The solubility behavior of polyamides was tested in common organic solvents such as amide-type, sulfone-bearing, phenol-like and furan-containing solvents, as shown in Table 1, especially with fluorine-containing polyamide **5e**, which displayed excellent solubility in common organic solvents and even in less polar solvents such as THF. As a result, there was an extra offering of the hexafluoroisopropylidene (-C(CF_3_)_2_-) segment in the polymer backbone. Apart from the more rigid backbone of polyamide **5b** derived from terephthalic acid, the excellent solubility of all polyamides can be apparently attributed to the incorporation of a flexible phenoxy linkage in the polymer main chain together with the bulky pyrene pendant group in the polymer backbone.

### 3.4. Thermal Properties

**Figure 5:**
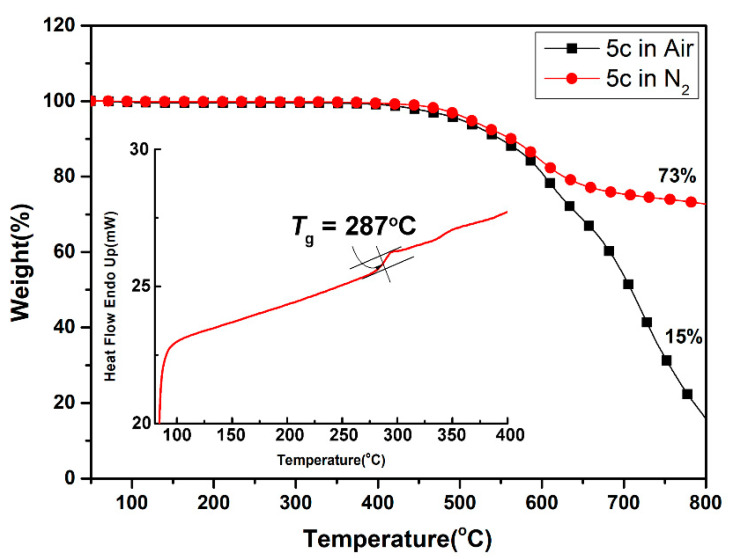
Representative TGA and DSC traces of polyamide **5c**.

For universal applications of these polymers in optoelectronics, the thermal stability and phase transition temperatures of polyamides were recorded using thermogravimetric analysis (TGA) and differential scanning calorimetry (DSC), and all the thermal behavior data of the polymers are summarized in Table 2. A representative set of TGA and DSC curves of polyamide **5c** are presented in Figure 5. Except for the semi-aromatic polyamide **5a** with aliphatic cyclohexane moiety, all the polymers possess excellent thermal stability and did not show significant weight loss up to temperatures of approximately 450 °C in both a nitrogen and air atmosphere. The decomposition temperatures (*T*_d_) at a 10% weight-loss of the aromatic polyamides (**5b** to **5e**) in nitrogen and air were recorded in the ranges of 544–562 °C and 519–549 °C, respectively. Except for aliphatic-aromatic polyamide **5a**, the amount of carbonized residue (char yield) of the other polyamides was more than 69% and as high as 74% at 800 °C in nitrogen. The high char yields of these fully aromatic polyamides can be ascribed to their high rigid aromatic composition. Due to the incorporation of thermally stable and the highly aromatic content of pyrene unit, all the polymers exhibited higher *T*_d_ values compared to their corresponding 5′ series analogue derived from 2,3-bis(4-aminophenoxy)naphthalene [49]. These thermal stable polyamides also have high glass-transition temperatures (*T*_g_) with a range from 276 to 310 °C. As expected, the lowest *T*_g_ of polymer **5a** can be illustrated in terms of the bendable polycyclohexane segments in its backbone. As compared to the naphthalene-based 5′ series analogs, the present polyamides 5 series demonstrates a remarkably increased *T*_g_ owing to the presence of rigid pyrene segments. Notably, among the substituent effects of these polyamides, it was found that the factors of the increased *T*_g_ values were dependent on the rigidity of the diacid counterpart structures. Thus, the thermal analysis reports above disclosed that these polyamides, especially the aromatic ones, exhibited excellent thermal stability, which, in turn, is beneficial to increase the manufacturing process in as optoelectronic device application and improve the morphological stability to both the spin-coated and slot-die coated film.

### 3.5. Photophysical Properties

**Figure 6:**
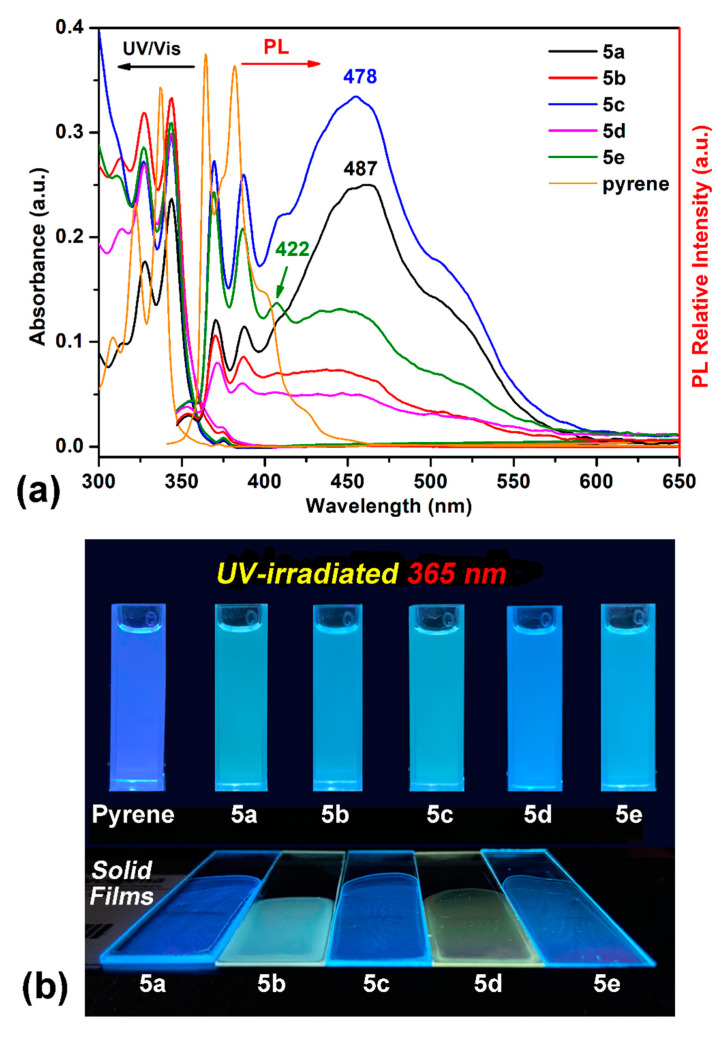
(**a**) Absorption and emission spectra of selected polyamides and pyrene in NMP solution (10 μM). (**b**) Photograph images were taken under illumination both of the dilute solutions and polymer films of a 365-nanometer UV light.

Due to the unique photophysical behaviors of polycyclic aromatic hydrocarbons with pyrene unit, all the dietherpyrene-based polyamides were detected using UV–Vis absorption and fluorescence spectroscopy in both solution and the solid film. Figure 6 shows the dilute polyamides **5a**–**5e** and pyrene in NMP solutions with absorptions and emission profiles together with their emission photograph images on exposure to an UV irradiation in solution and thin film on quartz plates. All of the UV–Vis absorption and fluorescence data are summarized in Table 3. All the polyamides displayed three obviously characteristic bands with an unsymmetrical shape and adjoining shoulders, the same as the pyrene’s absorption in the UV–visible region. The distinct high-energy absorption bands located at 344 nm are dominated to those emerging from the pyrene-based characteristic π-π* transition.

**Figure 7:**
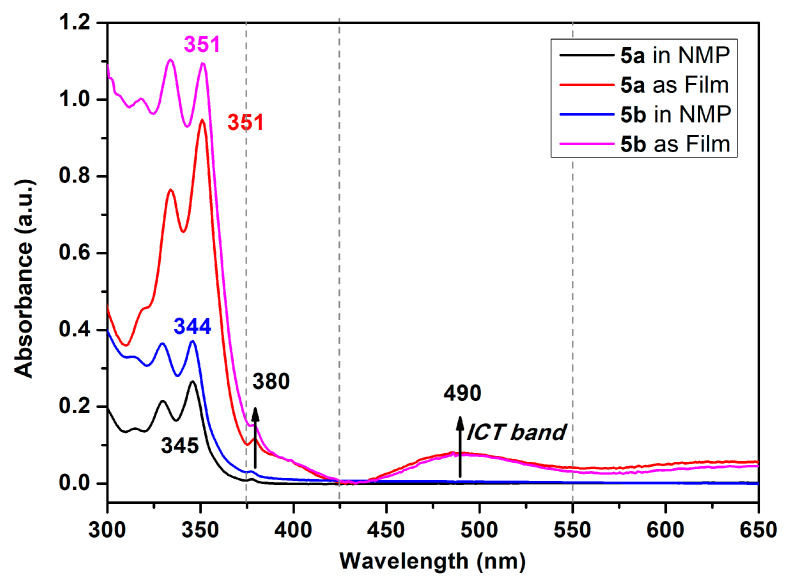
Absorption spectra of polyamides **5a** and **5b** in NMP solution (10 μM) and solid films coated on quartz glass.

These absorption bands of polyamides were similar to pyrene with a very slight redshift of ca. 7 nm, which, as a result of close packing from the polymer segment, intertwined with another. All the polyamides demonstrated similar absorption in NMP solutions the same as the pyrene’s spectra. Focusing on the absorption bands of polyamide both in NMP solutions and their solid film, as shown in Figure 7, the semi-aromatic polyamide **5a** and fully aromatic **5b** films were similar to their NMP solutions but their absorption intensities were a little bit different between 375 and 425 nm with a small shoulder centered at 380 nm with reasonable results from the efficient packing of the polymer chain. It is notable that the most bathochromically shifted transitions, located at a longer wavelength, which was centered at 490 nm, may be attributed to that coming from the phenoxy to pyrene and/or amide intramolecular charge-transfer (ICT) states as a mechanism similar to phenoxy-labeled perylenediimide derivatives [50]. Interestingly, the fluorescence emission profiles of these polyamides were dramatically different with pyrene. The polyamides **5** series revealed cognate emission contours in NMP solutions bearing particular pair sharp and single broaden peaks with emission maxima in the range 380~490 nm. This phenomenon clearly indicates that the emission in this system of polymers originates from different excited states due to the origin of the pyrene excimer formation compared with a single pyrene component. Whether an excimer formation arising in a given pyrene-containing system is readily determined by their broad emission featuring a center at ca. 480–500 nm is remarkably easy to identify, even when numerous pyrene monomer emissions take place, since the characteristic fluorescence pair emissions occurs in the 380–400 nm wavelength range [17]. As illustrated in Figure 6, the emission color of the polymer NMP solutions is sky blue, whereas that of non-substituted bare pyrene is deep blue.

As well-organized in Table 3, the fluorescence quantum yield (*ϕ*_F_) of these polymers in NMP solution were found from 0.10 to 0.50% relative to the 9,10-diphenylanthracene standard in cyclohexane. All the polyamides with a relatively low fluorescence quantum yield (*ϕ*_F_) may be attributed to the low energy excimer formation of pyrene rising from intra- and interchain charge transfer (CT) complexing between the high energy pyrene donor and the aryl amide (or aroyl) acceptor. Polyamides derived from aromatic dicarboxylic acid segments with the diphenyl ether and 2,2-isohexafluoropropane fragments seemed to have inhibited CT interactions and revealed a higher *ϕ*_F_, relative to as low as polyamide **5b** and **5d**. The CT-inhibited semi-aromatic polyamide **5a** derived from aliphatic dicarboxylic acid also showed a high *ϕ*_F_ of ca. 0.41%, similar to **5c** (0.5%). In addition to this, a strong interaction between the polyamide chain and amide-type NMP solvent may enhance the relaxation of the pyrene molecule in its excited state and lead to a reduced fluorescence quantum yield. The notable characteristic fluorescence features of pyrene and its excimer emission integration area ratio are listed in Table 3; we found that the chain length and polarity of the diacid segment strongly leads to a direct correlation with the extent of pyrene–pyrene intra- and interactions (with an order of **5a** > **5c** > **5e**), which, as a result of the emission properties, are finely dependent on the different diacid substituent patterns in the polymer backbone.

**Figure 8:**
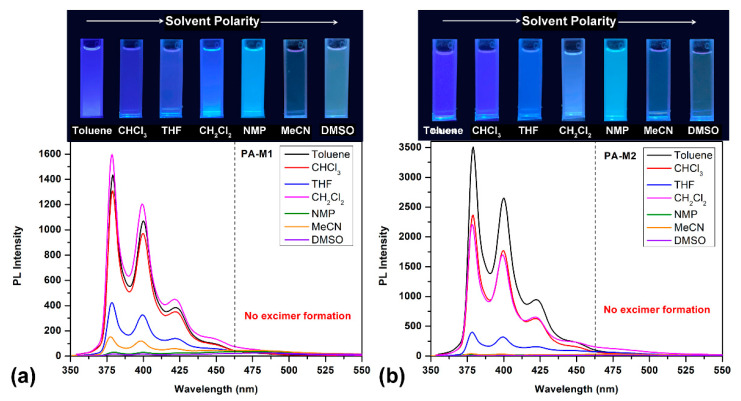
**PL** spectra of the dilute solution of model compound (**a**) **M1** and (**b**) **M2** (ca. 10 μM) in solvents of various polarity. Photographs were taken under illumination of a 365-nanometer UV light.

It is well known that solvatochromic dyes such as pyrene derivatives exhibited surrounding sensitive solvatochromic behavior in which the relative intensity of emission bands is dependent on solvent polarity [51]. Therefore, to further inquire into the solvatochromic properties of diphenoxypyrene-based polyamides, we evaluated the absorption and fluorescence emission of two amide-type model compounds, **M1** and **M2**, in the different polarity solvents. The model compound was selected for investigation because it exhibits better solubility than high molecular weight polymers; it can easy dissolve well in less polar solvents, such as toluene, chloroform and dichloromethane. Figure 8 shows the PL spectra of **M1** and **M2** in dilute solution in various solvents and is accompanying with the fluorescence images of its solutions.

Tables S1 and S2 were detailed descriptions of the absorption and fluorescent emission data of **M1** and **M2**, respectively. The solution absorption spectra of compound **M1** have two similar absorption bands (absorption λ_max_: 325~330 and 341~346 nm). However, the emission profile of compound **M1** in different solvents has the same emission bands located at ca. 380, 400 and 420 nm with different fluorescence intensities. Compared with the polyamides, the PL profile of model compound **M1** does not show the excimer formation around the 480-nanometer region; we attributed this phenomenon to the polymer chain self-intertwined leads that lock excimer formations. Interestingly, in the emission profile of the model compound in some solvents containing functional groups such as -O- for THF, -NHCO- for NMP and -SO_2_- for DMSO, their emission spectra show deep blue fluorescence emission with relative low intensity as compared with less polar solvents such as toluene, dichloromethane and chloroform. This phenomenon can be attributed to the fact that higher polar solvents are more likely to interact strongly with the amide functional groups on the polymer segment, resulting in a lower fluorescence intensity with a low fluorescence quantum yield. The absorption and emission data of model compound **M2** were similar to those of **M1** but had a higher fluorescence quantum yield and emission intensity due to the intramolecular CT inhibited from the aliphatic carboxylic acid group also listed in Table S2.

### 3.6. Electrochemical Data of Polyamides

**Figure 9:**
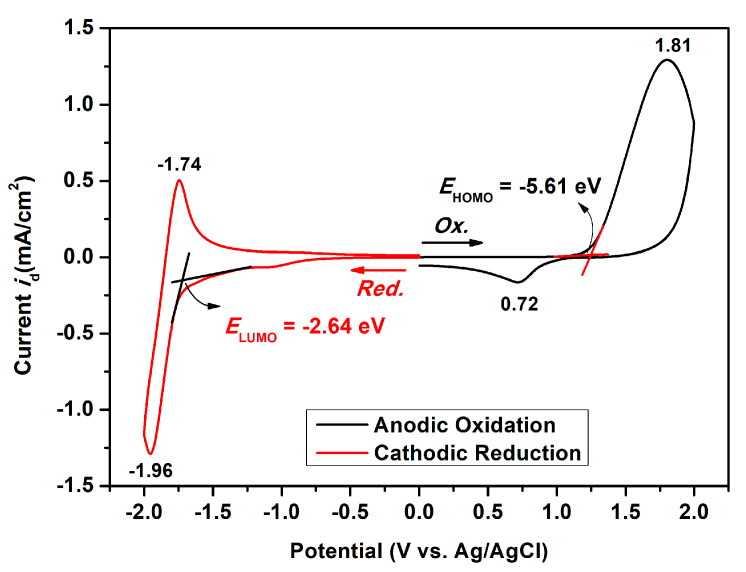
Cyclic voltammograms of the polyamide **5a** film on ITO-coated glass substrate in 0.1 M Bu_4_NClO_4_/CH_2_Cl_2_ (for oxidation trace) and DMF (for reduction trace) solutions at a scan rate of 50 and 100 mV/s, respectively.

**Scheme 4:**
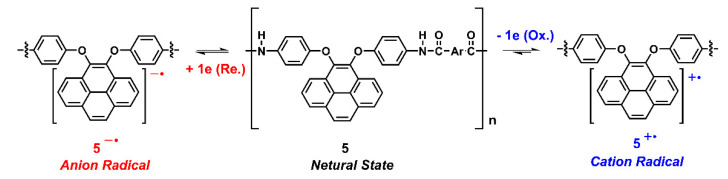
Presumed redox chemistry of dietherpyrene-containing polyamides.

The electrochemical properties of the dietherpyrene-cored polyamides were traced using cyclic voltammetry (CV) with a commercial available three-electrode system. The redox cycles of the polyamide-coated ITO glass samples were measured in 0.1 M Bu_4_NClO_4_/dichloromethane or acetonitrile and DMF, for the oxidation and reduction process, respectively. Figure 9 represented the typical CV curves of polyamide **5a** at a scan rate of 50 and 100 mV/s in its redox cycles. Polyamides 5 series exhibited an irreversible oxidation and a reversible reduction process at their first cycle, as shown in Figure 9 and Figure S13. The irreversible and reversible waves represented the formation of an unstable radical cation and a stable radical anion for pyrene originating from redox reactions of the dioxypyrene, as seen in the Scheme 4.

The onset potential (*E*^onset^) and the half-wave potentials (*E*_ox_^1/2^) of polyamides **5a**–**5e** under the oxidation process were recorded in the ranges from 1.20 to 1.25 V and 1.21 to 1.27 V, respectively (Table 4). The oxidation potentials are comparatively insensitive to the structure of the dicarboxylic acid unit in these polyamides. Variations of less than 0.06 V were observed in the **5a**–**5e** series, and this represents that the diacid part does not seriously affect the electronic nature of the oxidizable dietherpyrene core. The onset potential of the reduction process and half-wave potentials (*E*_red_^1/2^) of polyamides **5a**–**5e** were recorded in the range from −1.34 to −1.72 V and −1.47 to −1.99 V, respectively. In particular, the polyamide **5d** had two half-wave potentials (*E*_red_^1/2^) −1.47 and −1.93 V due to the sulfone (-SO_2_-) reduction to the sulfide (-S-) group of the diacid segment.

**Figure 10:**
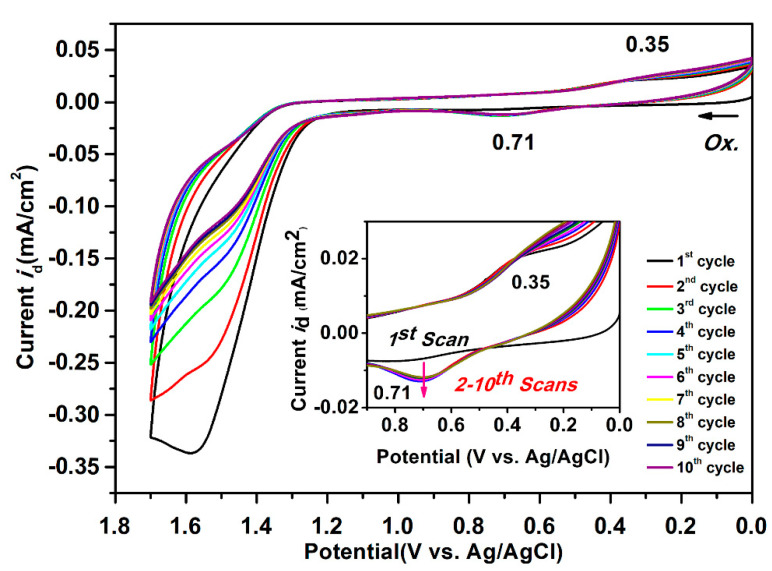
Cyclic voltammograms of 500 μM of the model compound **M2** in 0.1 M Bu_4_NClO_4_/CH_2_Cl_2_ (for the oxidation process) solutions at a scan rate of 50 mV/s.

**Scheme 5:**
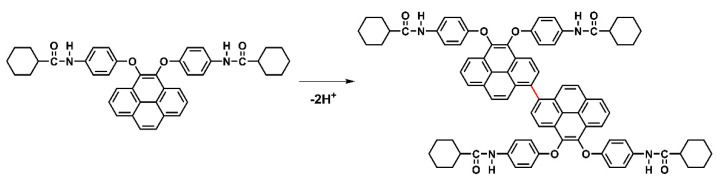
Proposed structures of electro-generated dimers of model compound **M2**.

Based on the oxidation and reduction onset potential of CV, the electrochemically bandgaps (*E*_g_^CV^) for polyamides **5a**–**5e** were determined in the range from 2.56 to 2.97 eV. The optical bandgaps (*E*_g_^opt^) calculated from the absorption edge (λ_onset_) of the polymer films were recorded in the range from 3.35 to 3.40 eV. Compared to *E*_g_^CV^ and *E*_g_^opt^, the bandgaps calculated from CV measurements were much lower than those obtained from the absorption spectra at about 0.33 to 0.74 eV. The highest occupied molecular orbital (HOMO) levels for the polyamides **5a**–**5e** were calculated and found to be between −5.56 and −5.61 eV and the values for the lowest unoccupied molecular orbital (LUMO) levels between −2.64 and −3.02 eV by using the oxidation and reduction onset potential. When the calculations were based on the *E*_1/2_ values, the HOMO and LUMO energy levels were estimated in the range of −5.53 to −5.59 eV and −2.37 to −2.89 eV, respectively. The CV curves of model compounds of **M1** and **M2** were similar as the polyamides, as shown in Figure S14, but the model compound **M1** did not find any anodic peak upon the oxidation process. These CV curves of the polyamides **5a**–**5e** with continuous ten cycles of a scan rate of 50 mV/s were similar to the model compound **M2** as shown in Figure 10. As shown in the first CV scan, the dioxypyrene unit underwent oxidation at about 1.3 V, followed by fully oxidation peak at 1.6 V. In the second scan, a new oxidation peak appeared at around 0.71 V and intensified the same peaks after ten cycles, which indicated the occurrence of the coupling reactions between two pyrene cation radicals forming the dimer-like bispyrene moiety in the 1- or 8-position of the electroactive site of the pyrene unit, as shown in Scheme 5 of the proposed structures of the electro-generated dimers.

**Figure 11:**
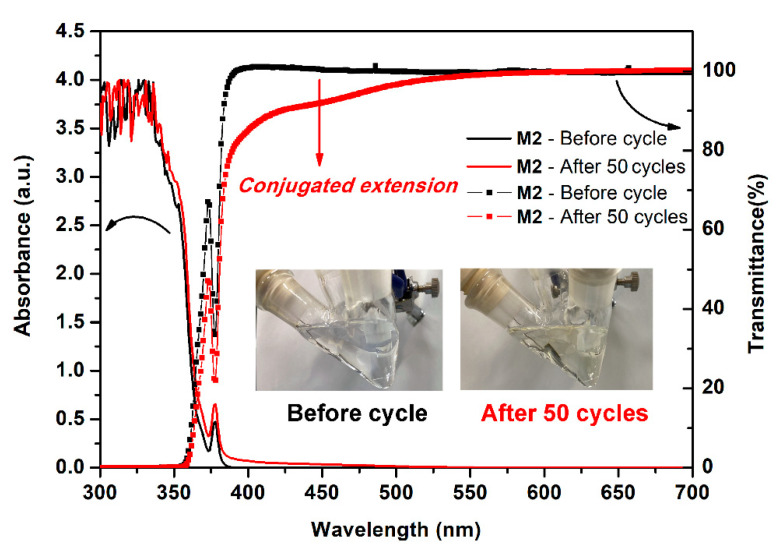
Absorption, transmittance spectra and inset images of the 500 μM model compound **M2** in 0.1 M Bu_4_NClO_4_/CH_2_Cl_2_ before and after continuous 50 cycles of CV test at a scan rate of 50 mV/s.

In order to confirm whether the phenomenon of dimerization occurred during the electrochemical process, we took continuous CV scans 50 times to monitor the change of the absorption and transmittance spectra of the **M2** containing electrolyte. As shown in Figure 11, the absorption between 375 to 500 nm was intensified gradually and more obviously to see the huge change of the transmittance in this region together with the color change of **M2** containing electrolytes from colorless to light yellow. Meanwhile, we attribute this spectral change to the formation of the bispyrene moiety that leads to an increased conjugated extension of high rigid aromatic content. According to these considerable and tunable HOMO and LUMO energy values of the optoelectronic polyamides, it might have potential use both as a luminescent and hole-transporting materials in OLEDs.

## 4. Conclusions

In this research, a new dietherpyrene-cored diamine was successfully synthesized, then followed with various dicarboxylic acids to obtain electroactive polyamides via polycondensation. The introduction of the bulky 4,5-diphenoxypyrene unit into the polyamide backbone affords excellent solubility with high thermal stability. Due to the polymer chain self-intertwined in diluted solution, these polyamides revealed remarkable excimer emission via locking excimer formations compared with corresponding model compounds. Both p- and n-doping from the nature of pyrene, these polymers have suitable energy levels. Thus, these prepared polyamides are considered to be promising candidates as luminescent and hole-transporting materials for use in optoelectronic devices.

## Figures and Tables

**Table 1:** Inherent Viscosity ^a^ and Solubility Properties ^b^ of Polyamides.

Polymer Code	*η*_inh_ (dL/g)	Various Solvent ^c^					
		NMP	DMAc	DMF	DMSO	*m*-Cresol	THF
**5a**	0.61	++ (++)	++ (++)	++ (++)	++ (++)	++ (++)	+− (−)
**5b**	0.44	++ (++)	+− (++)	+− (++)	+− (++)	− (−)	− (−)
**5c**	0.70	++ (++)	++ (++)	++ (++)	+ (++)	++ (++)	+− (−)
**5d**	0.41	++ (++)	++ (++)	++ (++)	+ (++)	++ (++)	+− (−)
**5e**	0.64	++ (++)	++ (++)	++ (++)	+ (++)	++ (++)	++ (++)
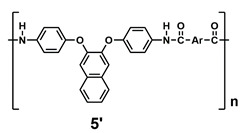							

^a^ Inherent viscosity tested at a concentration of 0.5 dL/g in DMAc at 30 °C. ^b^ Solubility: ++: soluble at room temperature; +−: partially soluble; +: soluble on heating; −: insoluble even on heating. ^c^ Solvent: NMP: *N*-methyl-2-pyrrolidone; DMAc: *N*,*N*-dimethylacetamide; DMF: *N*,*N*-dimethylformamide; DMSO: dimethyl sulfoxide; THF: tetrahydrofuran. Values in parentheses are data of analogous polyamides 5′ [49] with the same diacid residue as in the 5 series.

**Table 2:** Thermal behaviors of polyamides.

Polymer Code ^a^	*T*_g_^b^ (°C)	*T*_d_ 10 wt% Loss ^c^ (°C)		Char Yield ^d^ (%)
		In N_2_	In Air	
**5a**	276 (214) ^e^	485 (481) ^e^	482 (478) ^e^	55 (37) ^e^
**5b**	310 (255)	560 (517)	519 (504)	74 (69)
**5c**	287 (232)	562 (541)	549 (529)	73 (66)
**5d**	304 (267)	544 (505)	535 (497)	69 (63)
**5e**	294 (264)	555 (506)	547 (503)	69 (62)

^a^ The samples were heated at 300 °C for 1 h prior to the thermal analyses. ^b^ The film was heated from 50 to 400 °C at a scan rate of 20 °C/min followed by rapid cooling to 50 °C at −200 °C/min in nitrogen flow. The midpoint temperature of baseline shift on the follow-up DSC trace (from 50 to 400 °C at heating rate 20 °C/min) was defined as *T*_g_. ^c^ Decomposition temperature at which a 10% weight loss was recorded using TGA at a heating rate of 20 °C/min. ^d^ Residual weight percentages at 800 °C under nitrogen flow. ^e^ Values in parentheses are data of analogous polyamide 5′ having the same diacid residue as in 5 series.

**Table 3:** Photophysical behaviors of polyamides.

Polymer	In Solution ^a^				As Solid Film		
	λ_max_^abs^ (nm)	λ_max_^PL^ (nm) ^b^	*ϕ*_F_(%) ^c^	I_Pyr/Exc_^d^	λ_max_^abs^ (nm)	λ_onset_^abs^ (nm)	λ_max_^PL^ (nm) ^b^
**5a**	328, 345	381, 400, 487	0.41	0.13	334, 351	367	446
**5b**	327, 344	381, 400, 473	0.10	0.46	334, 351	370	465
**5c**	327, 344	380, 400, 478	0.50	0.21	334, 352	367	455
**5d**	328, 345	382, 399, 476	0.10	0.45	334, 351	370	473
**5e**	327, 344	380, 400, 475	0.23	0.78	332, 350	365	458

Notes: ^a^ The polymer concentration was 10 μM in NMP. ^b^ Excited at the absorption for both solution and solid film states. ^c^ Fluorescent quantum yield estimated by using 9,10-diphenylanthracene in cyclohexane (10 μM) as standard (*ϕ*_F_ = 90%). ^d^ Fluorescent emission integration area ratio (I) of characteristic pyrene (Pyr) and pyrene excimer (Exc). ^abs^: absorption. ^PL^: emission

**Table 4:** Redox potentials and energy levels of polyamides.

Polymer	Oxidation Potential (V) ^a^		Reduction Potential (V) ^b^			*E*_g_^CV^ (eV) ^c^		*E*_g_^opt^ (eV) ^d^	HOMO/LUMO (eV) ^e^	
	*E_ox_* ^onset^	*E*_ox_^1/2^	*E_red_* ^onset^	*E_red_* ^1/2^		*E* ^onset^	*E* ^1/2^		*E* ^onset^	*E* ^1/2^
				1^st^	2^nd^					
**5a**	1.25	1.27	−1.72	−1.85	−	2.97	3.08	3.38	−5.61/−2.64	−5.59/−2.51
**5b**	1.20	1.21	−1.64	−1.99	−	2.84	3.16	3.35	−5.56/−2.72	−5.53/−2.37
**5c**	1.21	1.25	−1.68	−1.86	−	2.89	3.07	3.38	−5.57/−2.68	−5.57/−2.50
**5d**	1.22	1.24	−1.34	−1.47	−1.93	2.56	2.67	3.35	−5.58/−3.02	−5.56/−2.89
**5e**	1.21	1.24	−1.68	−1.97	−	2.89	3.17	3.40	−5.57/−2.68	−5.56/−2.39

Notes: ^a^ vs. Ag/AgCl in acetonitrile or CH_2_Cl_2_. *E*^1/2^ = average potential of the redox couple peaks. ^b^ vs. Ag/AgCl in DMF. ^c^ Electrochemical bandgaps calculated from CV methods. ^d^ Optical bandgaps estimated from absorption edge of the film: *E*_g_^opt^ = 1240/λ_onset_. ^e^ The HOMO and LUMO energy levels were determined from *E*^onset^ and *E*^1/2^ values of CV curve and were referenced to ferrocene (4.8 eV relative to the vacuum energy level; *E*^onset^ = 0.44 V; *E*^1/2^ = 0.48 V). –*E*_HOMO_ = *E*^onset^ + 4.8–0.44 (eV); –*E*_HOMO_ = *E*^1/2^ + 4.8–0.48 (eV); –*E*_LUMO_ = –*E*_HOMO_–*E*_g_.

## Data Availability

The data presented in this study are available on request from the corresponding author.

## Supplementary Materials

The following supporting information can be downloaded at: https://www.mdpi.com/article/10.3390/polym14020261/s1, Figure S1: (a) ^1^H, (b) ^13^C NMR spectra of pyrene-4,5-dione in DMSO-*d*_6_; Figure S2: (a) H-H COSY and (b) C-H HSQC NMR spectra of pyrene-4,5-dione in DMSO-*d*_6_. Figure S3: (a) ^1^H and (b) ^13^C NMR spectra of dinitro compound 2 in DMSO-*d*_6_. Figure S4: (a) H-H COSY and (b) C-H HSQC NMR spectra of dinitro compound 2 in DMSO-*d*_6_. Figure S5: IR spectra of amide-type model compounds **M1** and **M2**. Figure S6: (a) ^1^H and (b) ^13^C NMR spectra of model compound **M1** in DMSO-*d*_6_. Figure S7: (a) H-H COSY and (b) C-H HSQC NMR spectra of model compound **M1** in DMSO-*d*_6_. Figure S8: (a) ^1^H and (b) ^13^C NMR spectra of model compound **M2** in DMSO-*d*_6_. Figure S9: H-H COSY spectra of model compound **M2** in DMSO-*d*_6_. Figure S10: C-H HSQC spectra of model compound **M2** in DMSO-*d*_6_. Figure S11: (a) IR spectra of polyamide **5e** thin film; (b) ^1^H and (c) H-H COSY NMR spectra of polyamide **5e** in DMSO-*d*_6_. Figure S12: WAXD patterns of polyamides **5a**-**5e**. Figure S13: Cyclic voltammograms of the polyamides (a) **5b**, (b) **5c**, (c) **5d** and (d) **5e** film on ITO-coated glass substrate in 0.1 M Bu_4_NClO_4_/CH_2_Cl_2_ or MeCN (only use in **5e**) (for the oxidation process) and DMF (for the reduction process) solutions at a scan rate of 50 and 100 mV/s, respectively. Figure S14: Cyclic voltammograms of 500 μM of model compound **M1** and **M2** in 0.1 M Bu_4_NClO_4_/CH_2_Cl_2_ (for oxidation) and DMF (for reduction) solutions at a scan rate of 50 and 100 mV/s, respectively. Table S1: photophysical properties of **M1** in different solvents. Table S2: photophysical properties of **M2** in different solvents. Scheme S1: Synthesis of amide-linkage model compound **M1** and **M2**.
