# Supplementary material for: Synthesis and Excimer Formation Properties of Electroactive Polyamides Incorporated with 4,5-Diphenoxypyrene Units
Source: Polymers (Basel). 2022 Jan 9;14(2):261. doi: 10.3390/polym14020261 (PMC8778140; doi:10.3390/polym14020261)
Supplement: Supplementary file 1 [file polymers-14-00261-s001.zip › polymers-1541125-supplementary.pdf]

Supplementary Materials

Synthesis and Excimer Formation Properties of Electroactive Polyamides Incorporated with 4,5-Diphenoxypyrene Units

Shih-Hsuan Chen, Huai-Sheng Chin and Yu-Ruei Kung *

Department of Chemical Engineering and Biotechnology, Tatung University, Taipei City 104327, Taiwan;
sean95123w@gmail.com (S.-H.C.); kim021324@gmail.com (H.-S.C.)
* Correspondence: yrkung@gm.ttu.edu.tw; Tel.:+886-2-77364660

1.1 Monomer Synthesis

1.1.1 Pyrene-4,5-dione (1)

The intermediate pyrene-4,5-dione was prepared using a modified method according to the literature. In a 1000-milliliter round-bottom flask equipped with an Olive-shaped magnetic stir bar, pyrene (10.0 g, 49 mmol) was dissolved in 200 mL of CH_2Cl_2 and 200 mL of MeCN. $NaIO_4$ (47.0 g, 220.5 mmol) was dissolved in 250 mL of warm water before $RuCl_3 \cdot$ x H_2O (1.0 g, 4.9 mmol) was added, and carefully added to the pyrene solution. The reaction mixture was vigorously stirred overnight at room temperature and the organic solvents were removed under reduced pressure. CH_2Cl_2 (200 mL) and H_2O (200 mL) were added to dissolve the solids, and the layers were separated. The aqueous phase was extracted with CH_2Cl_2 (5 × 100 mL), and the combined organic layers were washed with H_2O (5 × 100 mL), dried over anhydrous Na_2SO_4 or $MgSO_4$ and concentrated under reduced pressure to afford a dark orange solid (8.0g, 70%). The crude was purified using chromatography on silica gel using dichloromethane as the eluent to obtain pyrene-4,5-dione (**1**) as bright orange crystals (4.8 g, 60%). R_f (CH_2Cl_2) = 0.32, mp: 299–302 °C, measured using differential scanning calorimetry (DSC) at a scan rate of 10 °C/min.

IR(KBr): 1666 cm^{-1} (ketone C=O stretch). ^{1}H NMR (600 MHz, DMSO-d_6, δ, ppm): 8.33 (d, *J* = 7.9 Hz, 2H, H_d), 8.31 (d, *J* = 7.4 Hz, 2H, H_b), 8.01 (s, 2H, H_a), 7.83 (t, *J* = 7.7 Hz, 2H, H_c). ^{13}C NMR (150 MHz, DMSO- d_6, δ, ppm): 179.2 (C^7), 134.9 (C^5),131.4 (C^8), 130.5 (C^6), 128.6 (C^3), 127.9 (C^4), 127.7 (C^2), 127.1 (C^1).

*1.2.2 4,5-Bis(4-nitrophenoxy)pyrene (**2**)*

In a 500-milliliter round-bottom flask equipped with an Olive-shaped magnetic stir bar, a mixture of pyrene-4,5-dione (5.00 g, 21.5 mmol) and tetra-*n*-butylammonium bromide (*n*-Bu_4NBr) (6.90 g, 21.5 mmol) in 150 mL of tetrahydrofuran (THF) and 100 mL of H_2O was stirred under nitrogen and at room temperature for 30 min. After 30 min, sodium dithionite ($Na_2S_2O_4$) (37.40 g, 215 mmol) was added into the mixture and stirred for 30 min. Then, sodium hydroxide (10.44 g, 261 mmol) in 100 mL of H_2O was stirred for 30 min before adding 4-fluoronitrobenzene (20.0 g, 141 mmol) and heated to 80 ^{o}C. After stirring for 1 day, the reaction mixture was filtered, and it gave a brown solid. The brown solids were stirred in 200 mL of methanol; then, the yellow precipitates appeared. After, washed with a small amount of methanol and a large amount of water during the filtration process, it was taken it to dry at 80 ^{o}C in an oven. The dinitro compounds were recrystallized from glacial acetic acid with a yield of 77.7% (7.96 g) as fine yellow crystals (mp: 216-219 ^{o}C measured using differential scanning calorimetry (DSC) at a scan rate of 10 ^{o}C /min).

IR(KBr): 1589, 1338 cm^{-1} (asymm. and symm. -NO_2 str.), 1160 cm^{-1} (ether C-O str.). ^{1}H NMR (600 MHz, DMSO-d_6, *δ*, ppm): 8.49 (d, *J* = 7.6 Hz, 2H, H_d), 8.36 (s, 2H, H_a), 8.25 (d, *J* = 7.8 Hz, 2H, H_b), 8.17 (t, *J* = 7.7 Hz, 2H, H_c), 8.16 (d, *J* = 7.8 Hz, 4H, H_f), 7.16 (d, *J* = 9.3 Hz, 4H, H_e). ^{13}C NMR (150 MHz, DMSO-d_6, *δ*, ppm):

162.3 (C^9), 142.5 (C^7), 139.2 (C^{12}), 131.0 (C^8), 127.7 (C^1), 127.2 (C^4), 126.5 (C^5), 126.1 (C^{11}), 125.4 (C^2) 123.0 (C^6), 119.8 (C^3), 116.2 (C^{10}).

*1.1.3. 4,5-Bis(4-aminophenoxy)pyrene (**3**)*

In a 250-milliliter three-necked round-bottomed flask, 1.00 g of dinitro compound **2**, 0.05 g of 10 wt% Pd/C and 100 mL of ethanol were heated to reflux temperature with nitrogen atmosphere. To the mixture, 1.0 mL of hydrazine monohydrate was added slowly over a period of 10 min, then the solution was stirred at reflex temperature. After a further 3 h of reflux, the solution was filtered off Pd/C, and the filtrate was allowed to cool to room temperature to afford 0.52 g (59% yield) of diamine **3** as colorless to white rectangle-shaped crystals (mp: 215-217 ^oC measured using differential scanning calorimetry (DSC) at a scan rate of 10 °C/min).

IR(KBr): 3427, 3350 cm^{-1} ($-NH_2$ str.). ^1H NMR (600 MHz, DMSO-d_6, δ, ppm): 8.35 (d, J = 7.6 Hz, 2H, H_d), 8.26 (s, 2H, H_a), 8.26 (d, J =7.7 Hz, 2H, H_b), 8.08 (t, J = 7.7 Hz, 2H, H_c), 6.64 (d, J = 8.9 Hz, 4H, H_e), 6.45 (d, J = 8.9 Hz, 4H, H_f), 4.71 (s, 4H, $-NH_2$). ^{13}C NMR (150 MHz, DMSO-d_6, δ, ppm): 149.5 (C^{12}), 143.5 (C^7), 140.9 (C^9), 130.7 (C^8), 127.4 (C^1), 127.2 (C^2), 126.5 (C^4), 125.2 (C^5), 122.5 (C^6), 120.0 (C^3), 115.8 (C^{10}), 114.6 (C^{11}). Single crystal data: light-yellow rectangular prism crystal grown during slow crystallization in ethanol/THF (10:1 v/v), Crystal size: 0.52 mm × 0.49 mm × 0.23 mm, Monoclinic P 21/c with a = 15.2631(8) Å, b =7.0010 (3) Å and c = 18.9372(9) Å, α = 90°, β = 96.314(2)°, γ = 90°, where D_c = 1.375 Mg/m^3 for Z = 4 and V = 2011.30 (17) $Å^3$.

*1.1.4. 4,5-Di(4-benzamidophenoxy)pyrene (**M1**)*

A 50-milliliter round-bottom flask with a magnetic stirrer was charged with 0.416 g (1.0 mmol) of diamine monomer **3**, 0.244 g (2.0 mmol) of benzoic acid, 0.5 mL of triphenyl phosphite (TPP), 1.2 mL of NMP and 0.3 mL of pyridine. The reaction mixture was heated with stirring at 120 ^{o}C for 4 h. After reaction, the hot solution was poured into 200 mL of stirring methanol, giving rise to a white precipitate and placed overnight. Then, it was collected by filtration, washed thoroughly with hot water and methanol, respectively, to remove unreacted monomers, and dried in vacuo at 80 ^{o}C.

IR(KBr): 3336 cm^{-1} (amide N-H stretch), 1641 cm^{-1} (amide C=O stretch). ^{1}H NMR (600 MHz, DMSO-d_6, δ, ppm): 10.18 (s, 2H, amide N-H), 8.41 (d, J = 7.6 Hz, 2H, H$_d$), 8.31 (s, 2H, H$_a$), 8.27 (d, J = 7.9 Hz, 2H, H$_b$) , 8.13 (t, J = 7.7 Hz, 2H, H$_c$), 7.92 (d, J = 7.1 Hz, 4H, H$_g$), 7.65 (d, J = 9.1 Hz, 4H, H$_f$), 7.56 (t, J = 7.4 Hz, 2H, H$_i$), 7.51 (t, J = 7.8 Hz, 4H, H$_h$), 6.94 (d, J = 9.2 Hz, 4H, H$_e$). ^{13}C NMR (150 MHz, DMSO-d_6, δ, ppm): 165.3 (amide, C=O), 154.3 (C^{12}), 140.4 (C^{7}), 134.9 (C^{9}), 133.8 (C^{13}), 131.5 (C^{16}), 131.0 (C^{8}), 128.4 (C^{15}), 127.7 (C^{1}), 127.6 (C^{14}), 126.9 (C^{2}), 126.7 (C^{4}), 125.9 (C^{5}), 122.9 (C^{6}), 122.2 (C^{11}), 120.1 (C^{3}), 115.3 (C^{10}).

*1.1.5. 4,5-Di(4-cyclohexanecarboxamidophenoxy)pyrene (**M2**)*

Model compound **M2** was synthesized from the diamine monomer **3** and cyclohexanecarboxylic acid by a similar procedure as **M1** that was described above.

IR(KBr): 3295 cm^{-1} (amide N-H stretch), 2929, 2852 cm^{-1} (cyclohexyl C-H stretch) and 1651 cm^{-1} (amide C=O stretch). ^{1}H NMR (600 MHz, DMSO-d_6, δ, ppm): 9.69 (s, 2H, amide N-H), 8.39 (d, J = 7.7 Hz, 2H, H$_d$), 8.30 (s, 2H, H$_a$), 8.22 (d, J = 7.9 Hz, 2H, H$_b$), 8.10 (t, J = 7.7 Hz, 2H, H$_c$), 7.46 (d, J = 7.0 Hz, 4H, H$_f$), 6.84 (d, J = 7.0

Hz, 4H, H_e), 2.26 (t, *J* = 11.5 Hz, 2H, H_g), 1.61–1.76 (m, 10H, H_h+H_i+H_j), 1.15–1.41 (m, 10H, Hi+Hj). ^{13}C NMR (150 MHz, DMSO-d_6, δ, ppm): 173.8 (amide, C=O), 154.6 (C^{12}), 140.3 (C^7), 134.1 (C^9), 130.9 (C^8), 127.6 (C^1), 126.8 (C^4), 126.7 (C^2), 125.7 (C^5), 122.8 (C^6), 120.6 (C^{11}), 120.0 (C^3), 115.2 (C^{10}), 44.7 (C^{13}), 29.1 (C^{14}), 25.4 (C^{16}), 25.2 (C^{15}).

1.2 Direct synthesis of polyamides via the phosphorylation reaction

The synthesis of polyamide **5e** was used as an example to illustrate the general synthetic route used to produce the polyamides. A 50-milliliter round-bottom flask equipped with a magnetic stirrer was charged with diamine monomer **3** (0.3831 g, 0.92 mmol), 2,2-bis(4-carboxyphenyl)hexafluoropropane (**4e**) (0.3608 g, 0.92 mmol), 0.9 mL of TPP, 2.4 mL of NMP, 0.6 mL of pyridine and 0.15 g of dried calcium chloride ($CaCl_2$). Then, the mixture was heated with stirring at 115 °C for 4 h. After reacting for 4 h, the resulting polymer solution was poured slowly into 200 mL of stirring methanol, giving rise to a stringy, fiber-like precipitate, and was left to stand overnight. It was collected by filtration, washed thoroughly with hot water and methanol, and dried. The other polyamides were prepared using an analogous procedure.

IR (film): 3295 cm^{-1} (amide N-H stretch), 1627 cm^{-1} (amide C=O stretch). ^{1}H NMR (600 MHz, DMSO-d_6, *δ*, ppm): 10.33 (s, 2H, amide N-H), 8.36 (d, *J* = 7.5 Hz, 2H, H_d), 8.28 (s, 2H, H_a), 8.24 (d, *J* = 7.7 Hz, 2H, H_b), 8.09 (t, *J* = 7.7 Hz, 2H, H_c), 7.98 (d, *J* = 8.6 Hz, 4H, H_h), 7.63 (d, *J* = 9.1 Hz, 4H, H_f), 7.46 (d, *J* = 7.7 Hz, 4H, H_g), 6.93 (t, *J* = 9.1 Hz, 2H, H_e).

1.3. Preparation of Polyamide Films

A solution of polymer was made by dissolving about 0.7 g of the polyamide sample in 10–15 mL of hot DMAc with stirring. After completely dissolving, the homogeneous solution was poured into an 11-centimeter glass Petri dish that it was placed in an 80 °C oven overnight to remove most of the solvent of DMAc. Then, the semidried film was further dried in vacuo at 150 °C for 3 h. The obtained films were about 40–60 μm in thickness and were used for X-ray diffraction measurements, solubility test, thermal analyses and UV–PL spectroscopy.

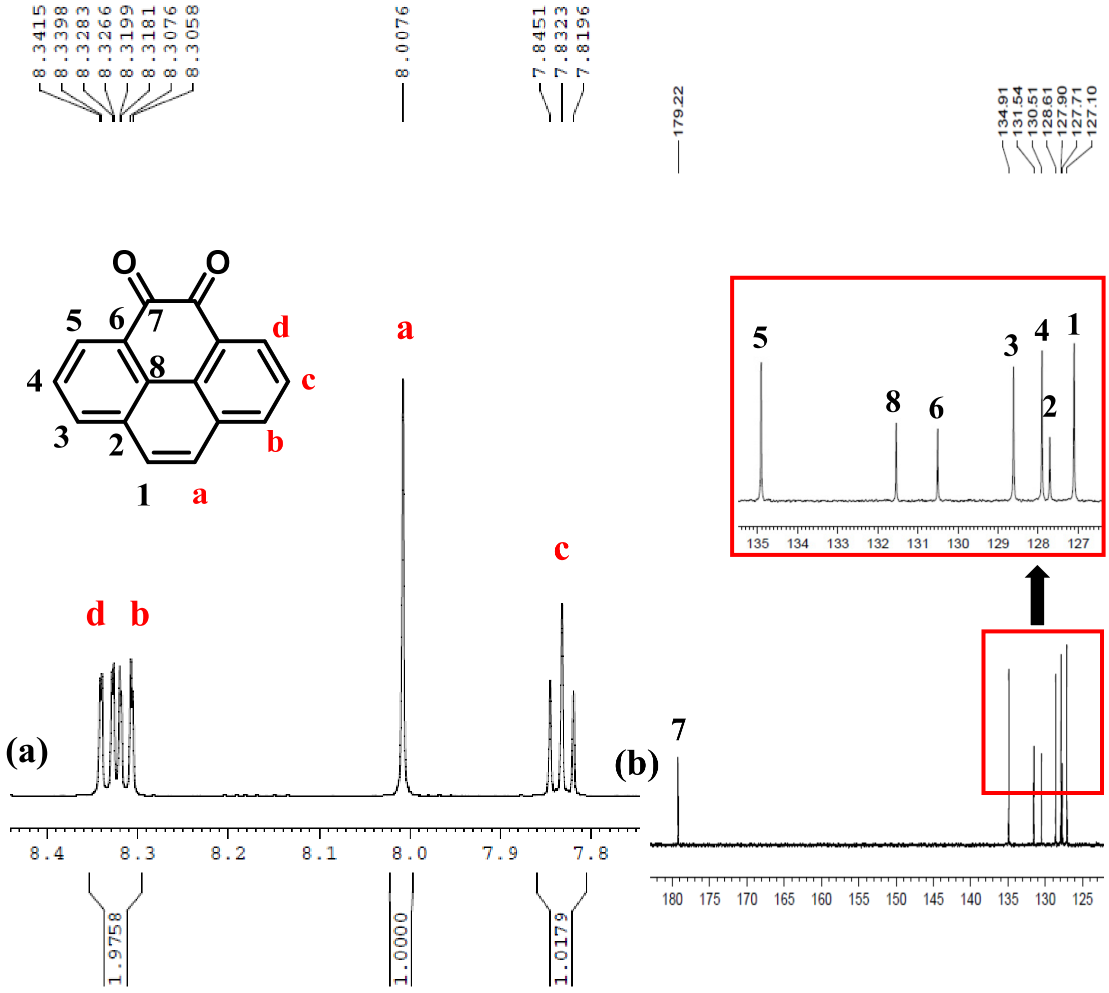

Figure S1 (a) ^{1}H, (b) ^{13}C NMR spectra of **pyrene-4,5-dione** in DMSO-d_6.

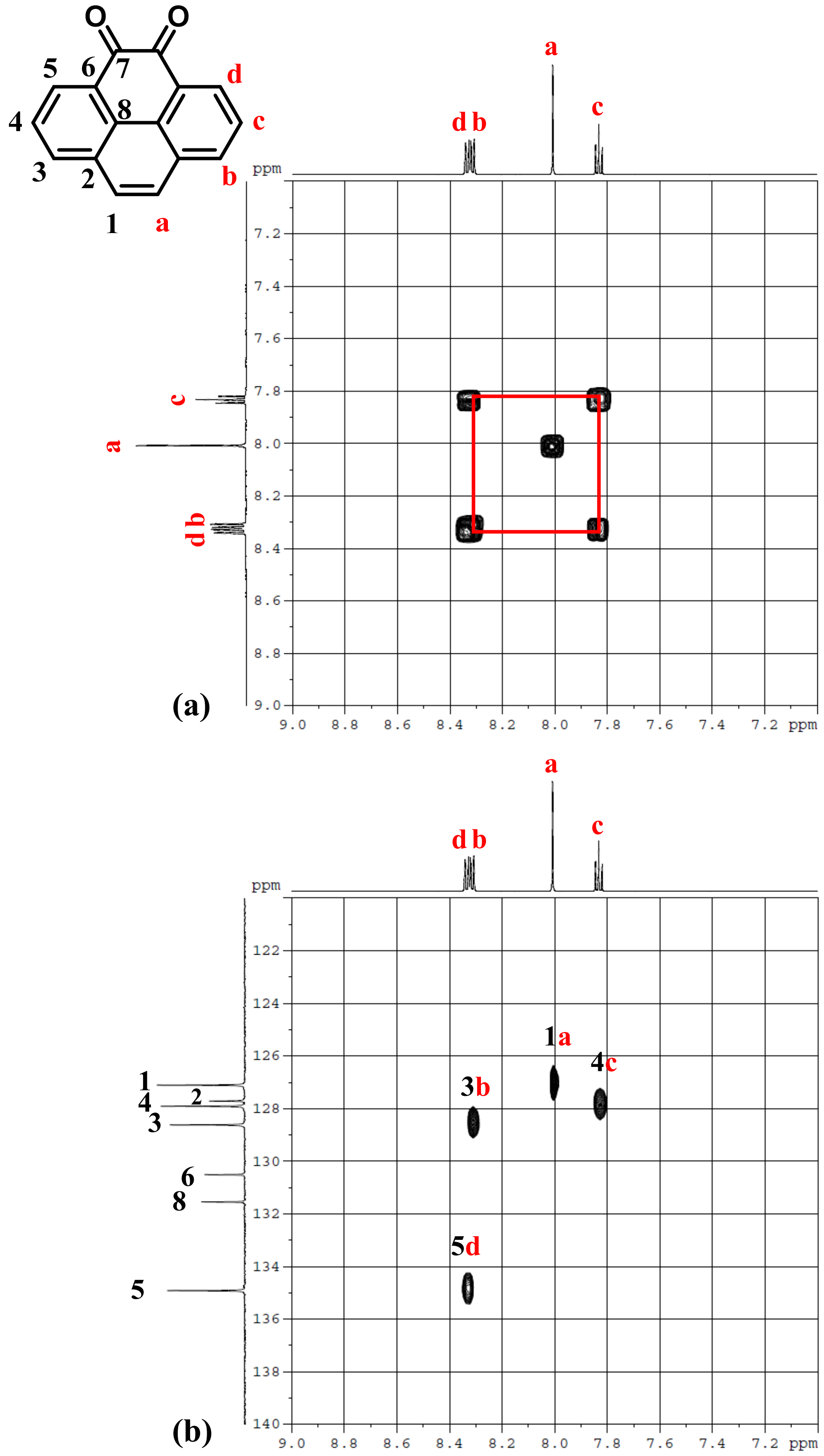

Figure S2 (a) H-H COSY and (b) C-H HSQC NMR spectra of **pyrene-4,5-dione** in DMSO-d_6.

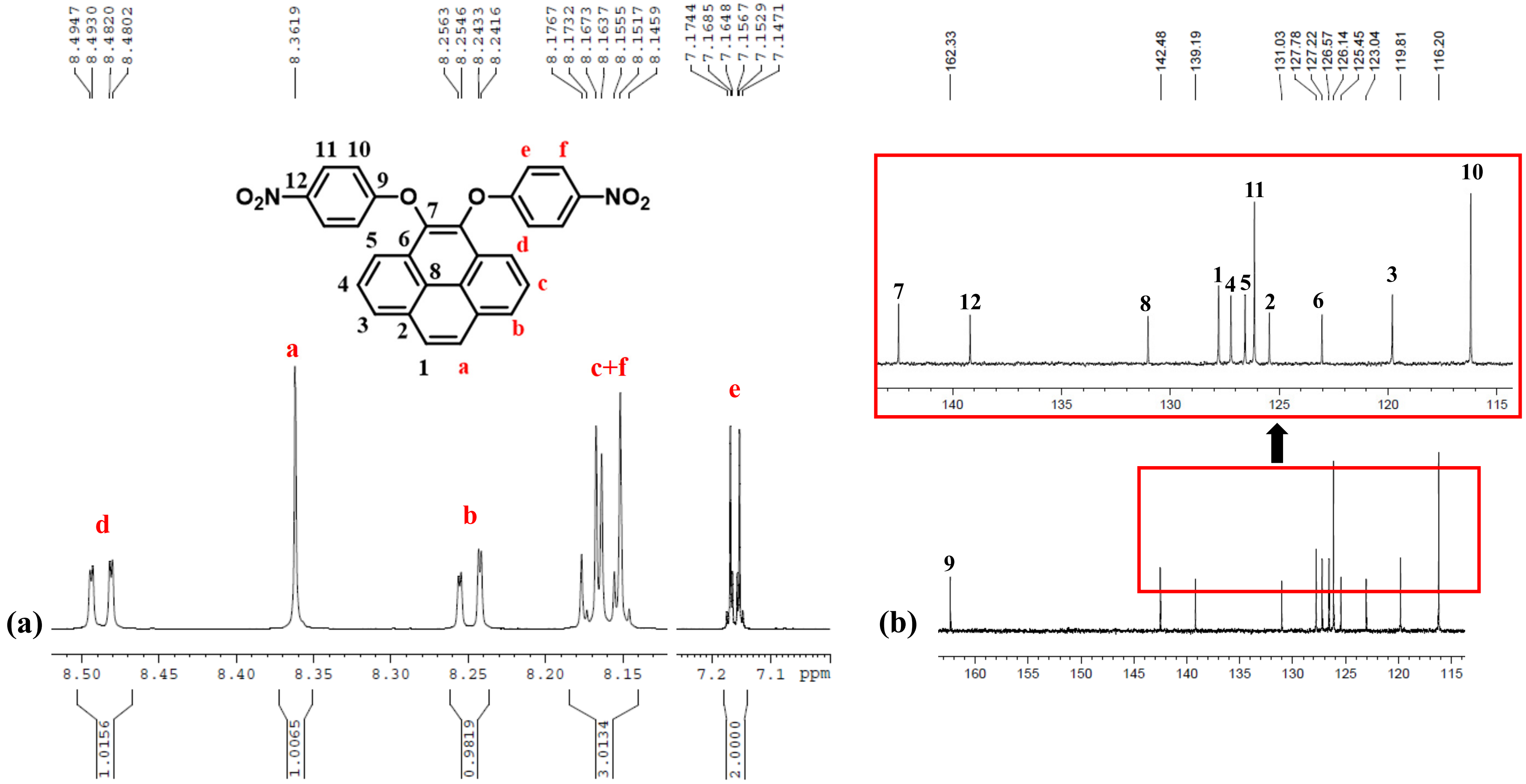

Figure S3 (a) ^{1}H and(b) ^{13}C NMR spectra of dinitro compound **2** in DMSO-d_6.

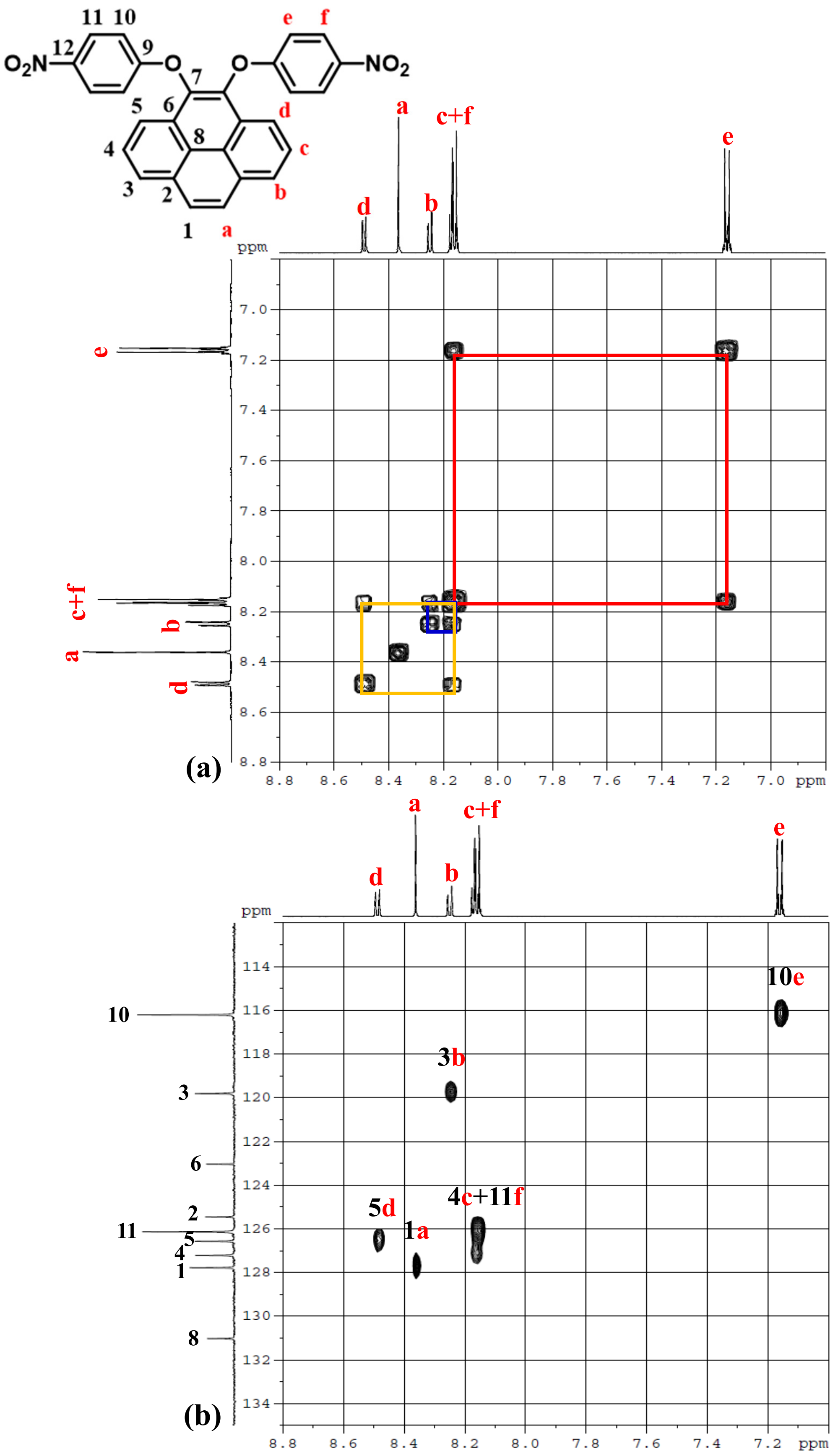

Figure S4 (a) H-H COSY and (b) C-H HSQC NMR spectra of dinitro compound **2** in DMSO-d_6.

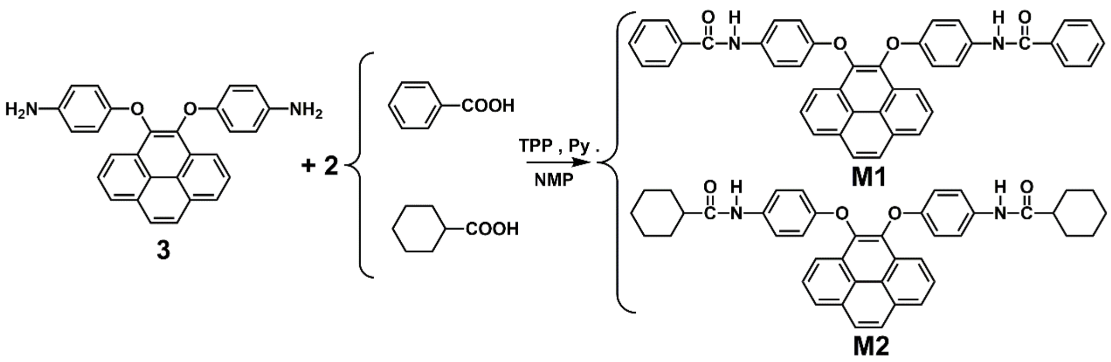

Scheme S1 Synthesis of amide-linkage model compound **M1** and **M2**.

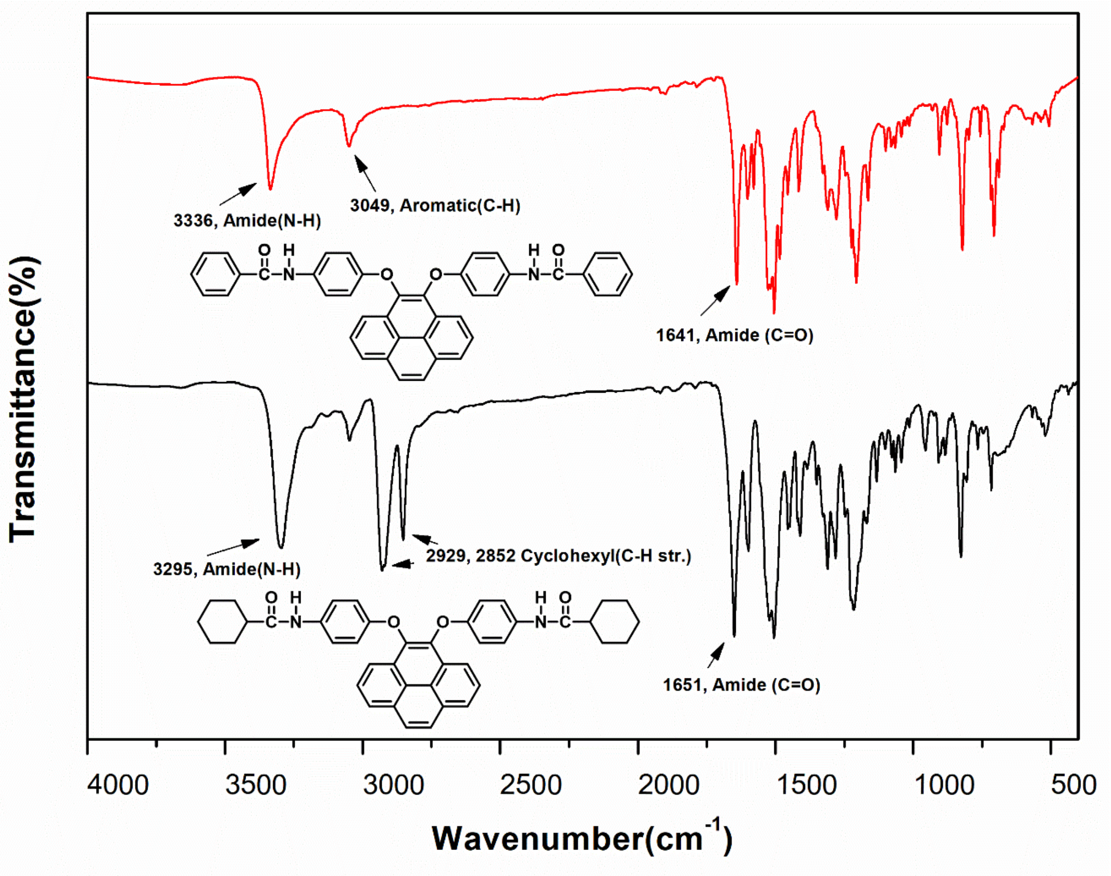

Figure S5 IR spectra of amide-type model compounds **M1** and **M2**.

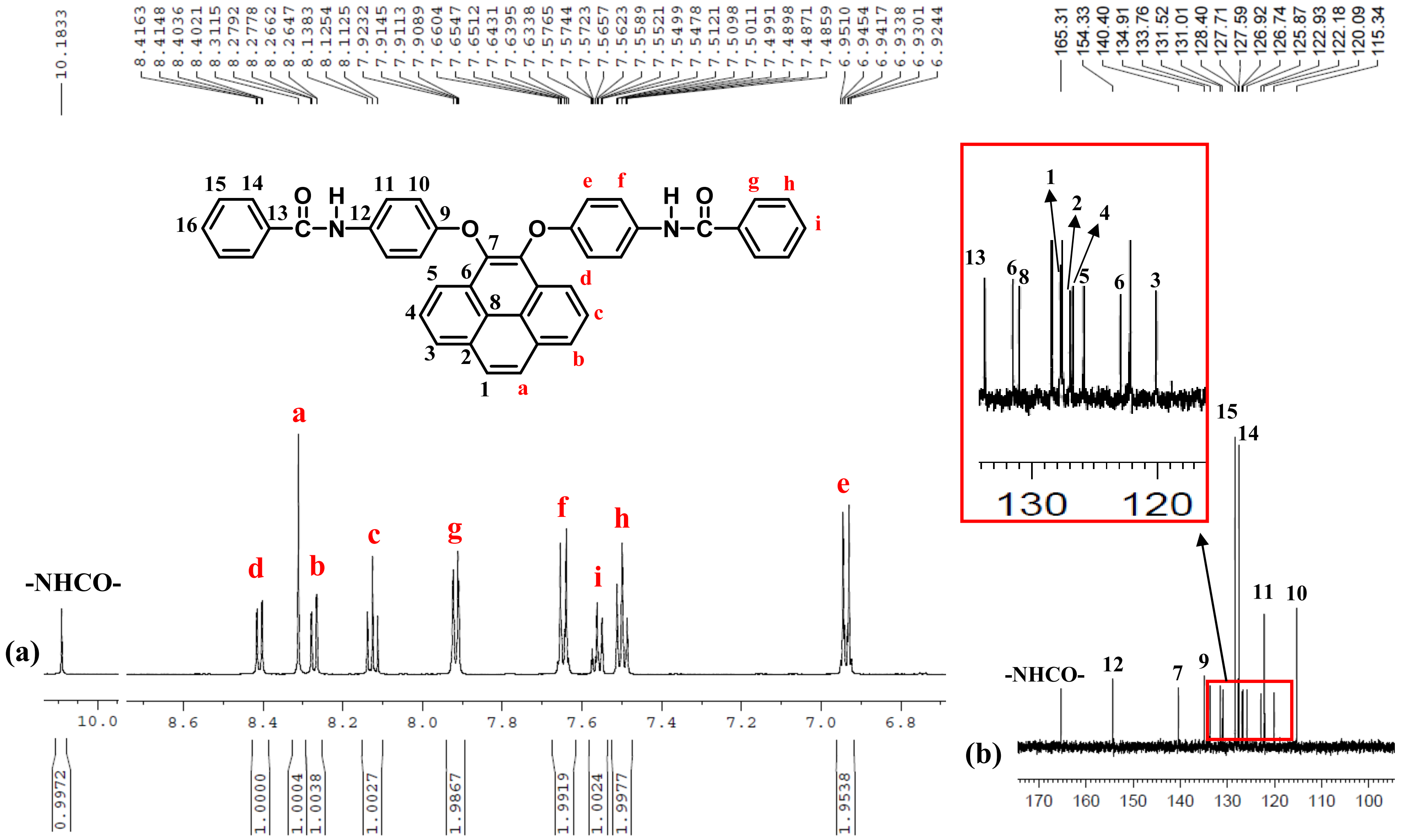

Figure S6 (a) ^{1}H and (b) ^{13}C NMR spectra of model compound **M1** in DMSO-d_6.

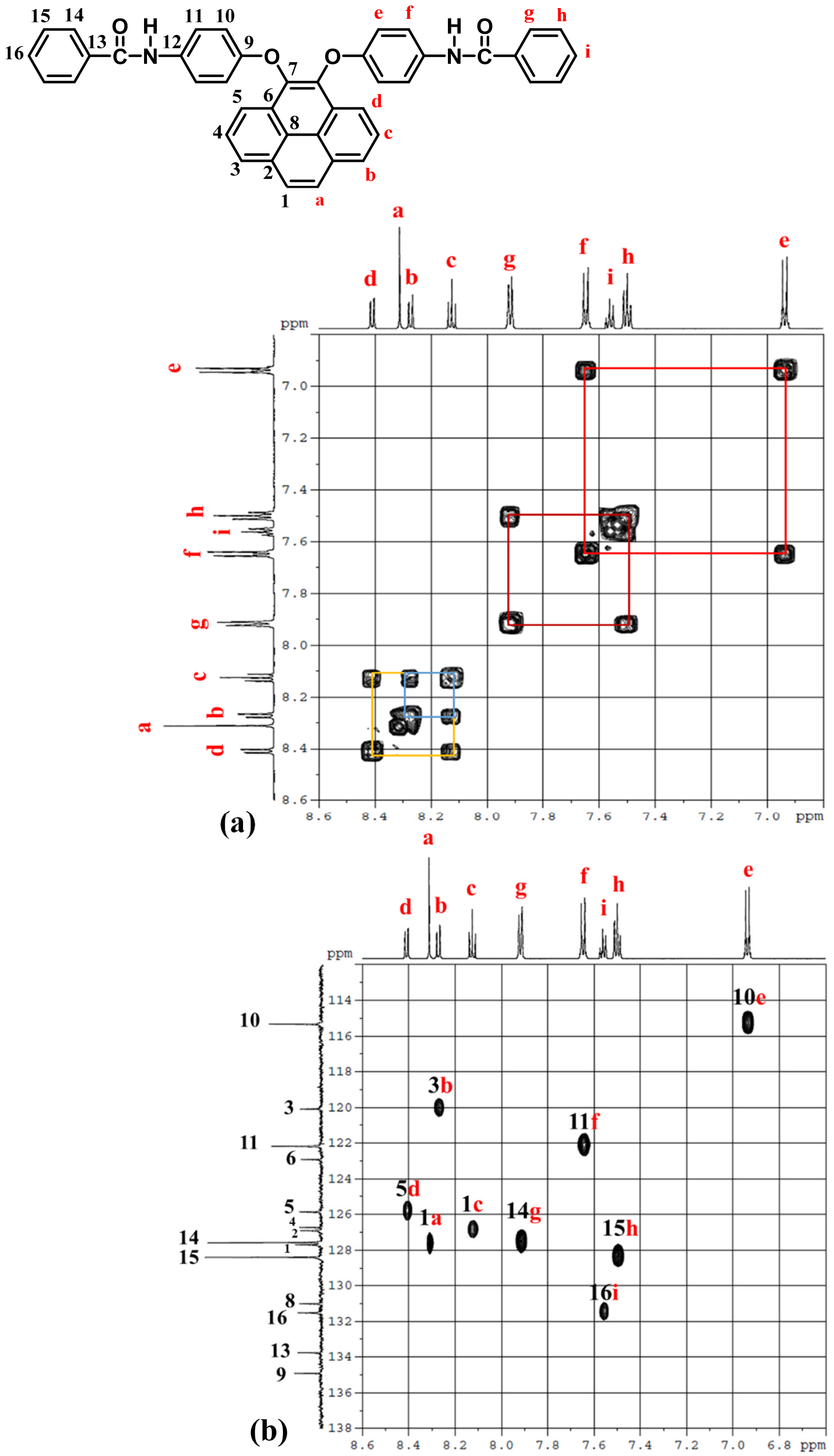

Figure S7 (a) H-H COSY and (b) C-H HSQC NMR spectra of model compound **M1** in DMSO-d_6.

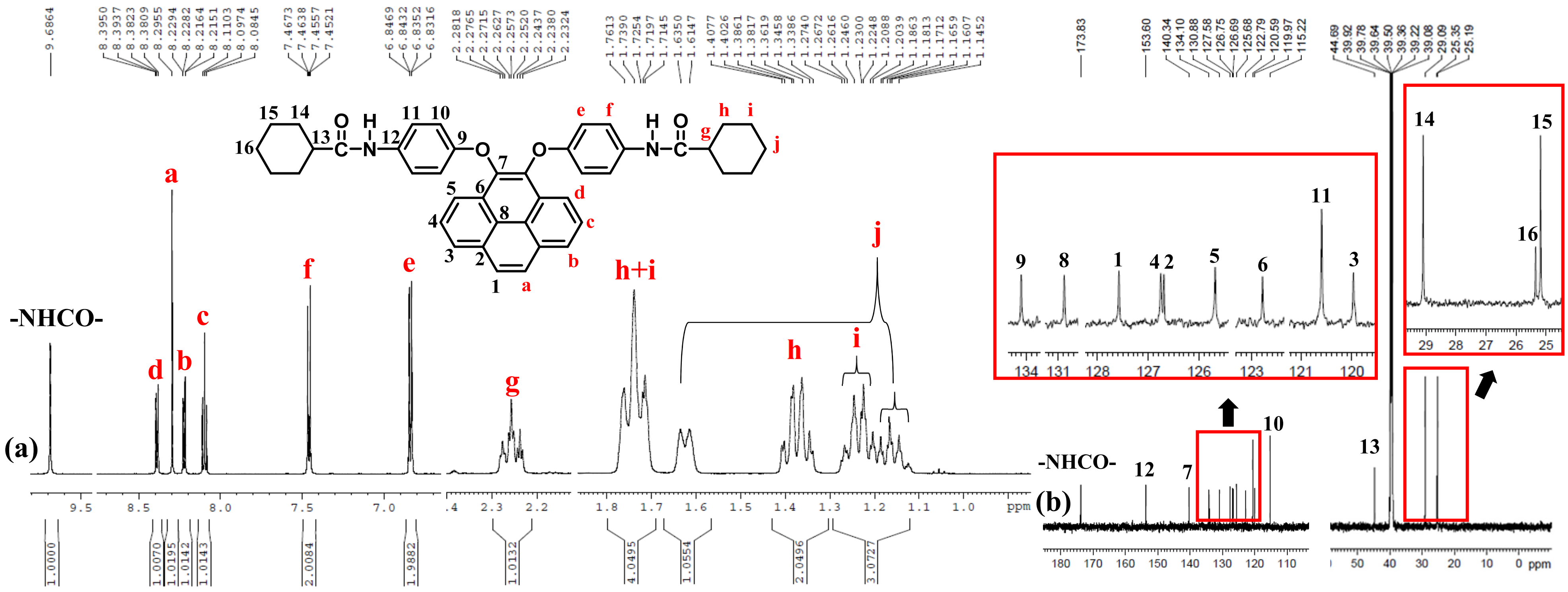

Figure S8 (a) ^{1}H and (b) ^{13}C NMR spectra of model compound **M2** in DMSO-d6.

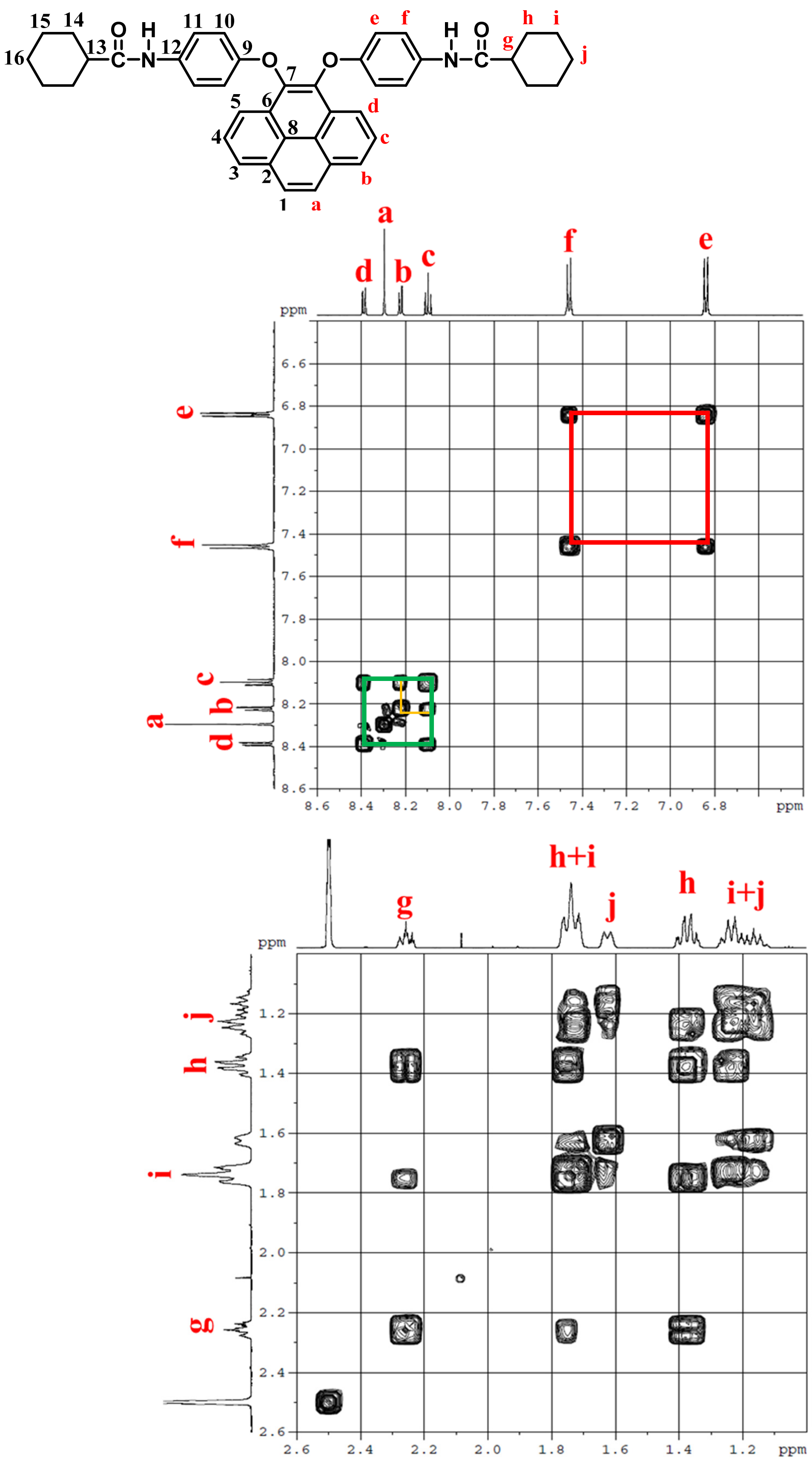

Figure S9 H-H COSY spectra of model compound **M2** in DMSO-d_6.

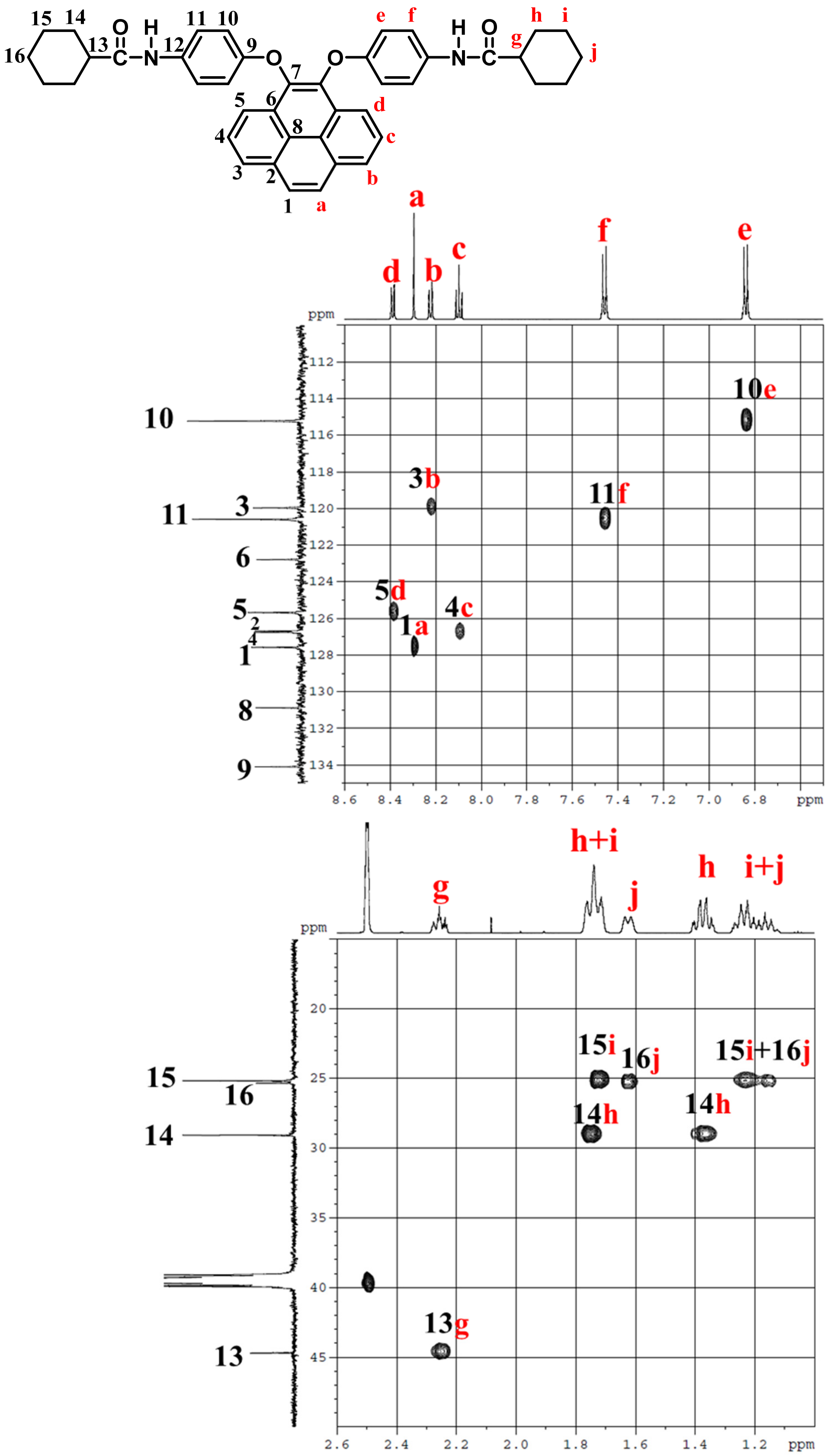

Figure S10 C-H HSQC spectra of model compound **M2** in DMSO-d_6.

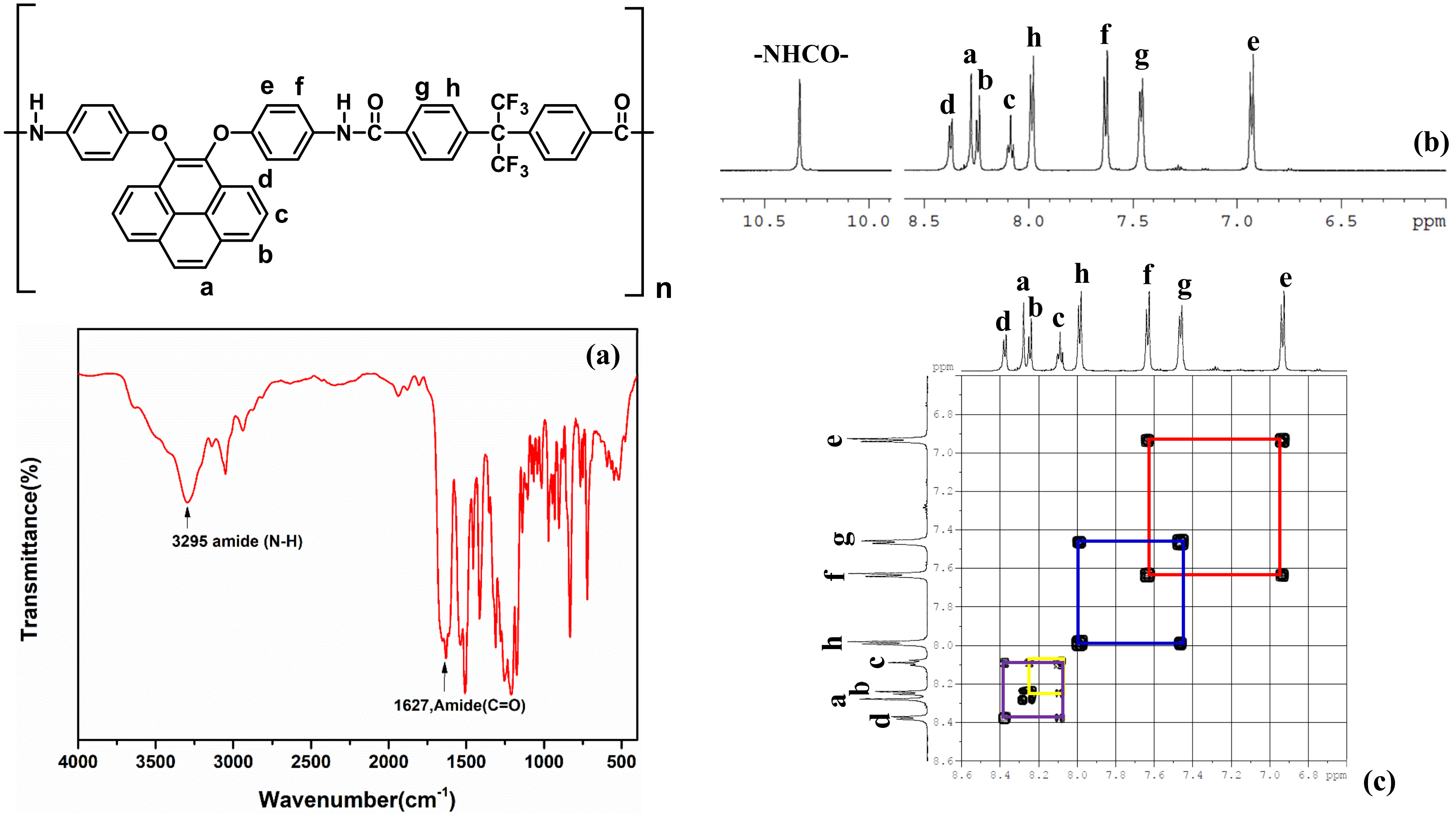

Figure S11 (a) IR spectra of polyamide **5e** thin film, (b) ¹H and (c) H-H COSY NMR spectra of polyamide **5e** in DMSO-d_6.

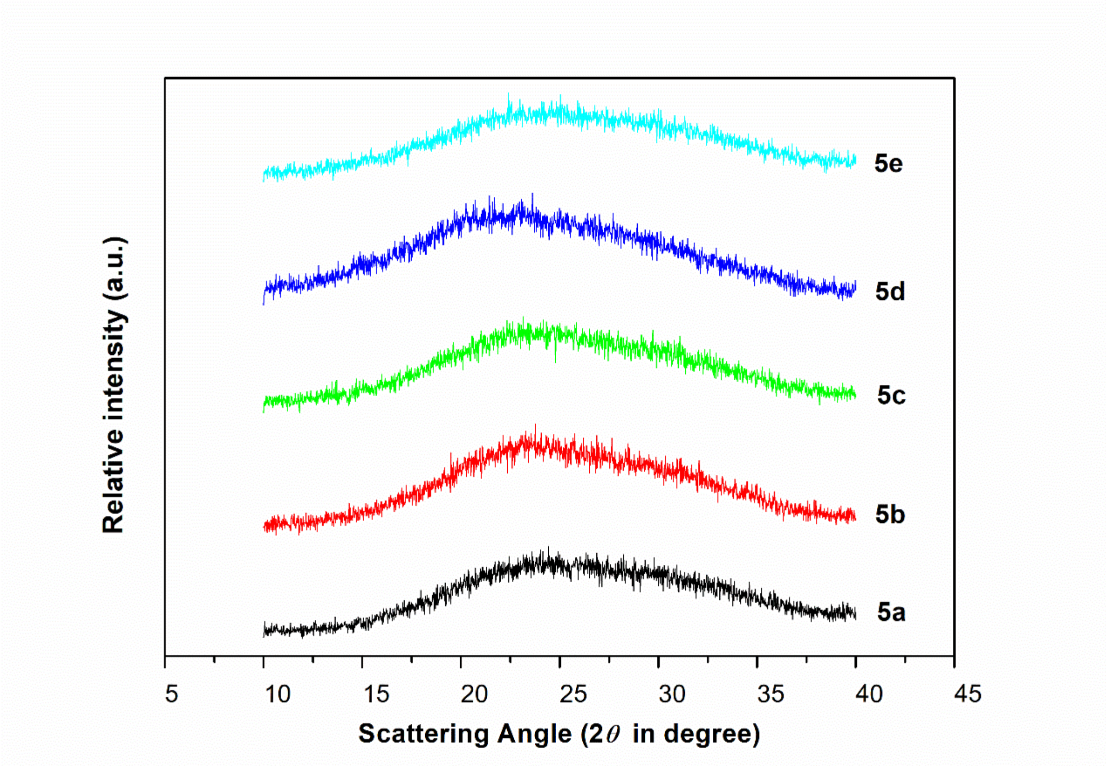

Figure S12 WAXD patterns of polyamides **5a–5e**.

Table S1 Photophysical Properties of **M1** in Different Solvents

Solvent	ε[a]	λ_{max}^{abs} (nm)[b]	λ_{max}^{PL} (nm)[c]	ϕ_F (%)[d]
**Toluene**	2.4	330, 346	379, 400, 423	3.9
**$CHCl_3$**	4.8	328, 344	379, 400, 422	4.0
**THF**	7.5	327, 343	379, 400, 422	1.5
**CH_2Cl_2**	9.1	328, 344	379, 400, 422	4.0
**NMP**	32.2	329, 345	380, 400, 473	0.5
**MeCN**	37.0	325, 341	378, 399, 421	0.7
**DMSO**	47.0	329, 345	380, 400, 487	0.4

[a] Dielectric constant of the solvent. [b] Concentration of the solution = 10 μM. [c] Excited at the absorption maximum. [d] Fluorescent quantum yield estimated using 9,10-diphenylanthracene in cyclohexane (10 μM) as standard (ϕ_F = 90%).

Table S2 Photophysical Properties of **M2** in Different Solvents

Solvent	ε[a]	$_{max}^{abs}$ (nm)[b]	$_{max}^{PL}$ (nm)[c]	ϕ_F (%)[d]
**Toluene**	2.4	329, 345	379, 400, 423	8.2
**$CHCl_3$**	4.8	328, 343	379, 400, 422	7.7
**THF**	7.5	326, 342	379, 400, 422	1.7
**CH_2Cl_2**	9.1	327, 343	379, 399, 422	6.3
**NMP**	32.2	328, 344	380, 399, 422	0.5
**MeCN**	37.0	324, 340	378, 399, 421	0.4
**DMSO**	47.0	328, 344	381, 401, 423	0.3

[a] Dielectric constant of the solvent. [b] Concentration of the solution = 10 μM. [c] Excited at the absorption maximum. [d] Fluorescent quantum yield estimated using 9,10-diphenylanthracene in cyclohexane (10 μM) as standard (ϕ_F = 90%).

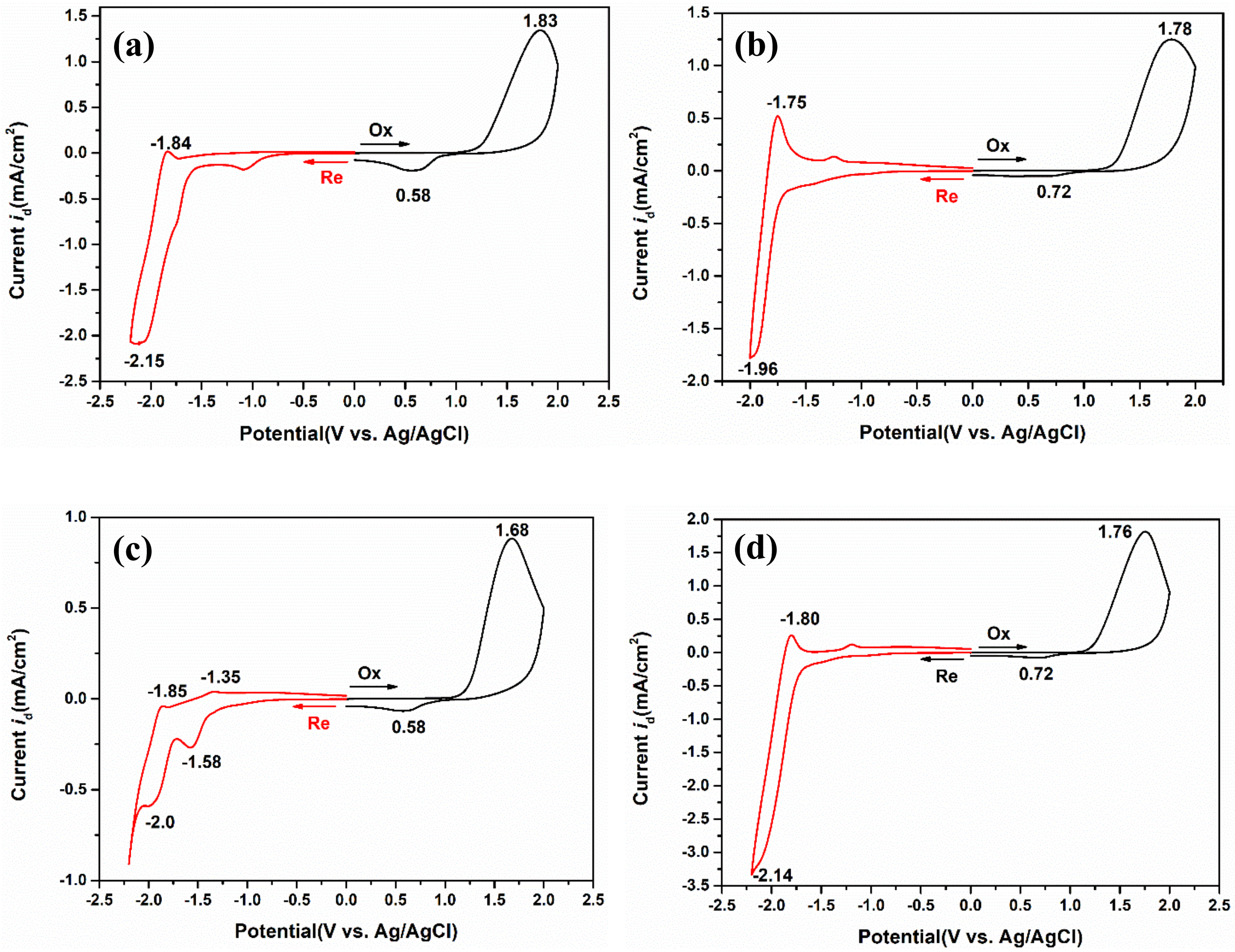

Figure S13 Cyclic voltammograms of the polyamides (a) **5b**, (b) **5c**, (c) **5d** and (d) **5e** film on ITO-coated glass substrate in 0.1 M Bu_4NClO_4/CH_2Cl_2 or MeCN (only use in **5e**) (for the oxidation process) and DMF (for the reduction process) solutions at a scan rate of 50 and 100 mV/s, respectively.

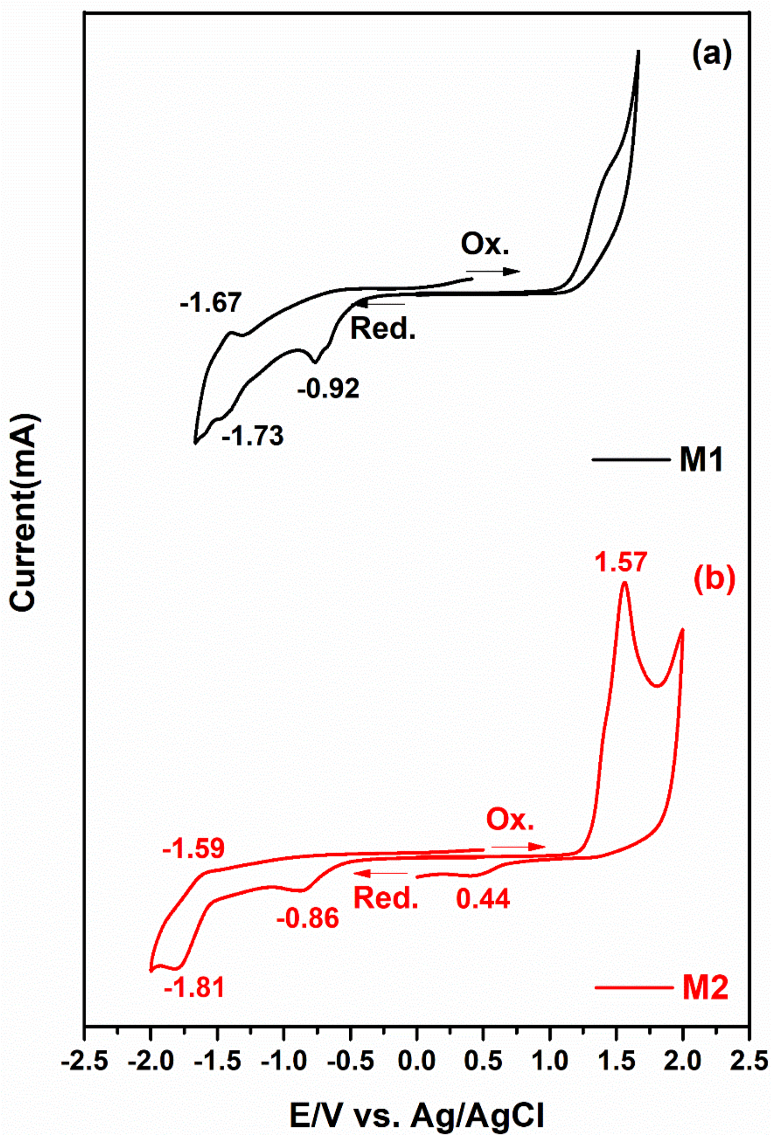

Figure S14 Cyclic voltammograms of 500 μM of model compound **M1** and **M2** in 0.1 M Bu_4NClO_4/CH_2Cl_2 (for oxidation) and DMF (for reduction) solutions at a scan rate of 50 and 100 mV/s, respectively.
